# Optimal tool design in micro-milling of difficult-to-machine materials

**DOI:** 10.1007/s40436-022-00418-w

**Published:** 2022-10-17

**Authors:** Lorcan O’Toole, Feng-Zhou Fang

**Affiliations:** 1 Centre of Micro/Nano Manufacturing Technology (MNMT-Dublin), University College Dublin, Dublin 4, Ireland; 2 State Key Laboratory of Precision Measuring Technology and Instruments, Laboratory of Micro/Nano Manufacturing Technology (MNMT), Tianjin University, Tianjin, 300072 People’s Republic of China

**Keywords:** Precision machining, Micro-milling, Difficult-to-machine (DTM), Tool wear, Micro-tool design, Surface quality

## Abstract

The limitations of significant tool wear and tool breakage of commercially available fluted micro-end mill tools often lead to ineffective and inefficient manufacturing, while surface quality and geometric dimensions remain unacceptably poor. This is especially true for machining of difficult-to-machine (DTM) materials, such as super alloys and ceramics. Such conventional fluted micro-tool designs are generally down scaled from the macro-milling tool designs. However, simply scaling such designs from the macro to micro domain leads to inherent design flaws, such as poor tool rigidity, poor tool strength and weak cutting edges, ultimately ending in tool failure. Therefore, in this article a design process is first established to determine optimal micro-end mill tool designs for machining some typical DTM materials commonly used in manufacturing orthopaedic implants and micro-feature moulds. The design process focuses on achieving robust stiffness and mechanical strength to reduce tool wear, avoid tool chipping and tool breakage in order to efficiently machine very hard materials. Then, static stress and deflection finite element analysis (FEA) is carried out to identify stiffness and rigidity of the tool design in relation to the maximum deformations, as well as the Von Mises stress distribution at the cutting edge of the designed tools. Following analysis and further optimisation of the FEA results, a verified optimum tool design is established for micro-milling DTM materials. An experimental study is then carried out to compare the optimum tool design to commercial tools, in regards to cutting forces, tool wear and surface quality.

## Introduction

Micro-milling is one of the most cost effective and flexible manufacturing processes to produce micro-components, micro-features and micro-structured surfaces as it provides relatively high material removal rates and allows for manufacturing of complex three-dimensional surfaces, while maintaining high precision and component accuracy. One of the earliest applications for the micro-milling process was in micro-structure and micro-component fabrication, which included blades of an impeller or turbine, walls of a microchannel, microcolumns, and fins of a heat exchanger. Presently, these microstructures have been widely applied in micro-fuel cells [1], microfluidic chip channels [2], electrical discharge machining (EDM) electrodes [3], microchannels for heat exchangers [4], and mass sensing in microelectromechanical system (MEMS) devices [5]. A key area for micro-milling is the mould making, which takes advantage of the high material removal rate to allow for cost effective manufacturing of moulds with high aspect ratio micro-structures. A clear example of the micro-milling efficiency is in the rapid prototyping of microfluidic moulds and devices [6]. Another major application of the micro-milling process is in micro-texturing and micro-patterning to reduce frictional forces and reduce wear between parts [7, 8], as well as increase lubricity [9], which will have great prominence in the near future of bio-implant manufacturing. However, the inherent issues of tool wear and poor machined surface quality in the micro-milling process currently reduce its effectiveness in such industries [10]. This is primarily true for machining of typical difficult-to-machine (DTM) materials commonly found in both mould and orthopaedic implant manufacturing [11]. Such DTM materials have properties of very high hardness and resistance to wear, which include cobalt-chrome alloys, titanium alloys, mould steels and ceramics. These material properties lead to high cutting forces, excessive tool wear, poor surface roughness and burr formation, as well as high cutting temperatures. Most importantly, however, premature failure and complete tool breakage of current commercially available fluted micro-milling tools is well known in industry, and is clearly a significant barrier facing the application of micro-milling for machining DTM materials in these industries.

The issues of poor rigidity, weak cross sections and geometries, fragile cutting lengths and overall poor mechanical strength all arise from the fact that current commercially available micro-milling tools are generally downscaled from macro-milling tools [12].The problem with this method of micro-milling tool design is mainly two-fold. Firstly, fluted macro-milling tools are designed for heavy machining with large material removal rates, where chip evacuation and removal of large and continuous chips is the most important. Flute depth is therefore a key design criterion of macro-milling tools to prevent chip packing during heavy material removal applications and during machining of ductile materials where long continuous chips form. Flutes in micro-milling tools are a less important design feature due to the relative size of the chips formed as a result of much higher spindle speeds and comparatively much lower depth of cuts. This results in smaller and discontinuous chip formation, which can be removed from the cutting zone directly by cutting fluid, rather than the necessity for the chip to traverse up and along a flute, away from the working zone. This is even more critical during micro-milling of very hard materials, where small and discontinuous chip formation is essential to limiting cutting force and ensuring efficient removal of heat from the working zone. The second and most important concern with downscaling macro-milling tools to micro-milling tools relates to the square-cube law; as shapes decrease in size, its volume shrinks faster than its surface area. This leads to the critical issue of weak cross sectional cutting lengths with large flute valleys in the tool, severely reducing tool stiffness and overall mechanical strength, while causing an increase of the tool deflection. All of which contribute to excessive tool wear, edge chipping and premature tool failure during micro-milling.

In order to combat the inherent issue of tool wear and poor surface quality during micro-milling of DTM materials, a fundamental investigation of the optimal tool geometry is presented in this study. Beginning with a design approach to micro-milling tool geometries, a novel tool design is established that focuses on alleviating the known issues of current tool geometries that lead to significant tool wear and tool breakage during machining of very hard materials. From the developed design, static stress and deflection finite element analysis (FEA) is carried out to identify stiffness and the maximum deflections as result of distributed applied loads, analogous to cutting forces during micro-milling. A fractional parametric experimental study was then carried out on the fabricated tools to compare the developed tool design with commercially available fluted tools, in regards to cutting forces, tool wear and surface quality. Finally, a tool wear criterion was developed to characterise the condition of micro-milling tools.

## Design approach

### State of the art

The reduced stiffness of commercial fluted tools often leads to large tool deflection, leading to high cutting forces and uneven chip loading, eventually resulting in tool chipping and tool breakage as well as significant tool wear. It was determined by Fang et al. [13] through a comprehensive study that two-flute end mills were 8–12 times weaker than the ∆-type and D-type end mills with a tapered body, while Fleischer et al. [14] determined that a larger bulk cross sectional area would impart higher stiffness into the tool. Similarly, more cutting flutes or a larger helix angle led to better tool stiffness, as determined by Shi et al. [15]. Finally, Cheng et al. [16] presented that tools with smaller rake angles of − 70° had better stiffness and lower tool wear rate based on their FEA simulations and experimental tests. It is also imperative to impart high strength in the micro-milling tool. Failure of the tool, such as edge chipping or tool breakage, is a significant issue which is initiated at specific weakened areas in the micro-tool and may occur as a result of tool run-out, tool deflection or chatter. The cutting edge corners are the most loaded part of the cutting edges, as described by Li et al. [17]. Generally, the cutting edge radius for common tungsten carbide micro-tools is between 0.8 µm and 5 µm owing to restraints of the tool fabricating process. Wu et al. [18] found that the cutting edge radius was the most influential factor on the tool’s process output performance. Another important specification of micro-tool design is to ensure that the tool has high durability, thereby increasing tool life and wear resistance. Micro-milling tools are generally manufactured from wear resistant and hard materials, such as tungsten carbide (WC), and diamond tools. However, the specification of the material will also highly influence the quality of the tool. Generally, sharper and more homogeneous cutting edges could be achieved with smaller grain sizes, as studied by Kirsch et al. [19]. The edge strength of the tool then depended on the cobalt binder phase, according to Zhan et al. [20]. Although diamond tools are regarded as a very suitable micro-milling tool material [19], the focus of this work is to optimise tool geometry, so fine-grain WC tools are used. A simplified and symmetrical geometry for ease of manufacturing and to maintain tool balance during very high spindle speeds is another fundamental specification of micro-milling tools. Due to the limitation of conventional tool manufacturing process such as grinding and wire electrical discharge machining (WEDM), it is difficult to achieve conformity and accuracy when the features are in the micro-domain in a cost effective and efficient manner [12]. Through FEA, Fleishcer et al. [14] considered a relatively symmetrical straight edge end-mill (SEE) with a single cutting edge of simplified geometry, while Cheng et al. [16] fabricated their SEE tool by only three linear and a half rotational computer numerical control (CNC) WEDM axis. Finally, another major issue of current micro-tool designs pertains to the formation of burrs, which is an accumulation of material to form raised edge or volume on the workpiece surface after machining. Burr formation was found to be influenced mostly by using multi-edge cutting tools [21]. Another way to minimise burr formation was to use a tapered cutting edge length [22]. Tool life will also be extended by reducing rubbing at non cutting edges and decreasing the pinching of formed chip between the tool and the workpiece surface, further improving the chip removal capability [23].

### Design specification

For micro-milling, appropriate geometrical design of the tool is essential for achieving robust tool stiffness and mechanical strength, to prolong tool life and ensure effective machining. Specifically, the geometry of micro-end mill tools should be designed depending on the type of applications, in this case, for machining of very hard and wear resistant materials. Therefore, this design specification outlines several key areas for improving overall tool stiffness, strength, durability, ease of fabrication and ensuring efficient chip formation and evacuation, while minimising contact between the tool and the workpiece. Therefore, the proposed tool is a micro double straight edge end-mill (DSEE) with the following criteria.

(i) High stiffness due to a large cross-sectional area along the cutting-edge length, with optimised tool features and angles including large taper angle neck, short cutting-edge length, short overall length, as well as optimised radial and axial, rake, and clearance angles.
(ii) High strength through increased material volume around the cutting edge radius and peripheral cutting edges, optimised tool edge radius and peripheral cutting edge radius, increased bulk cross section and reduced depth of cutting channels.
(iii) High durability due to the specification of cemented carbide tool material, such as fine grain WC.
(iv) A simplified, balanced and radially symmetrical geometry design with two straight cutting edges with dodecagon clearance faces to ensure that fabrication of the tool is both efficient and cost-effective.
(v) Efficient chip evacuation through optimised channel and cutting edge geometry with the addition of a chip breaker on the rake and flank face of the tool to prevent pinching and to direct chip flow radially inward to ensure small, discontinuous chip formation.

## FEA

The DSEE tools were designed using Autocad for tool diameters 800 µm, 600 µm, 400 µm and 200 µm. FEA was then conducted using Abaqus to study the linear, steady-state, static analysis of the distribution of the maximum principal stress and deflection of the cutting length under applied loads during micro-milling. The performances of both tools were investigated in relation to tool geometry, and the results from which led to further optimisation and then fabrication of the DSEE tool design.

### DSEE tool design

**Fig. 1:**
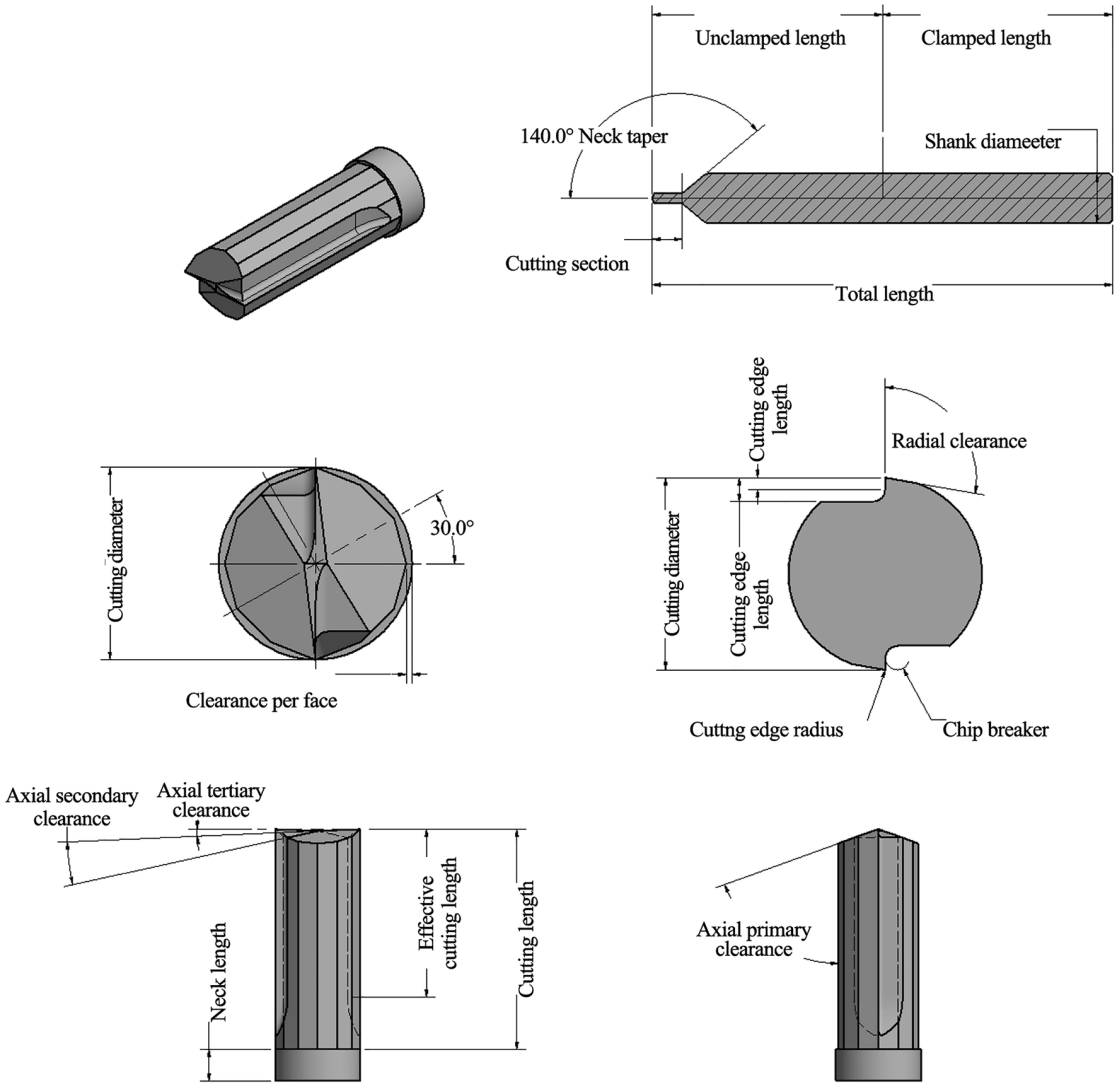
Schematic diagram of geometrical features of designed DSEE tool

Figure 1 shows both the overall geometrical dimensions of the DSEE designed micro-milling tool, as well as the clearance and chip breaker features. Table 1 details the dimensions for both tool diameters. To avoid contact between the radial flank face and the side wall of the workpiece during machining, the tools also have a radial clearance. The tools also have twelve faceted faces, known as dodecagon clearance faces, to again ensure there is no unwanted contact between the tool and workpiece, even at high feed rates. The cutting and peripheral faces are extended to outer diameter. The straight cutting edges with round edge radius direct chip flow radially inward to the centre of the tool and towards the chip breaker feature, which works to reduce chip sizes and prevent continuous chip formation.

**Table 1:** DSEE tool design dimensions and features

	Tool diameter/µm	
	800	600
Cutting diameter/mm	0.8	0.6
Cutting length/mm	2.100	1.575
Unclamped length/mm	18.5	18.5
Clamped length/mm	18.5	18.5
Effective cutting length/mm	1.6	1.2
Total length/mm	37	37
Cutting section length/mm	2.4	1.8
Neck length/mm	0.300	0.225
Cutting edge length/mm	0.100	0.875
Chip breaker length/mm	0.050 0	0.037 5
Shank diameter /mm	4	4
Clearance per face/mm	0.025 0	0.018 8
Chip breaker/mm	0.050 0	0.037 5
Cutting edge radius/mm	0.005	0.005
Axial primary clearance/(°)	70	70
Axial secondary clearance/(°)	2.5	2.5
Axial tertiary clearance/(°)	10	10
Radial clearance/(°)	100	100

### FEA model and procedure

The main objective of this FEA analysis is to evaluate the deflection and Von Mises stress distribution at the tool tip of the DSEE designed tool and compare the results with a typical, commercially available 2-flute micro-milling tool. The results led to further optimisation of the DSEE tool design, from which tools were then fabricated and a full experimental investigation was conducted.

**Fig. 2:**
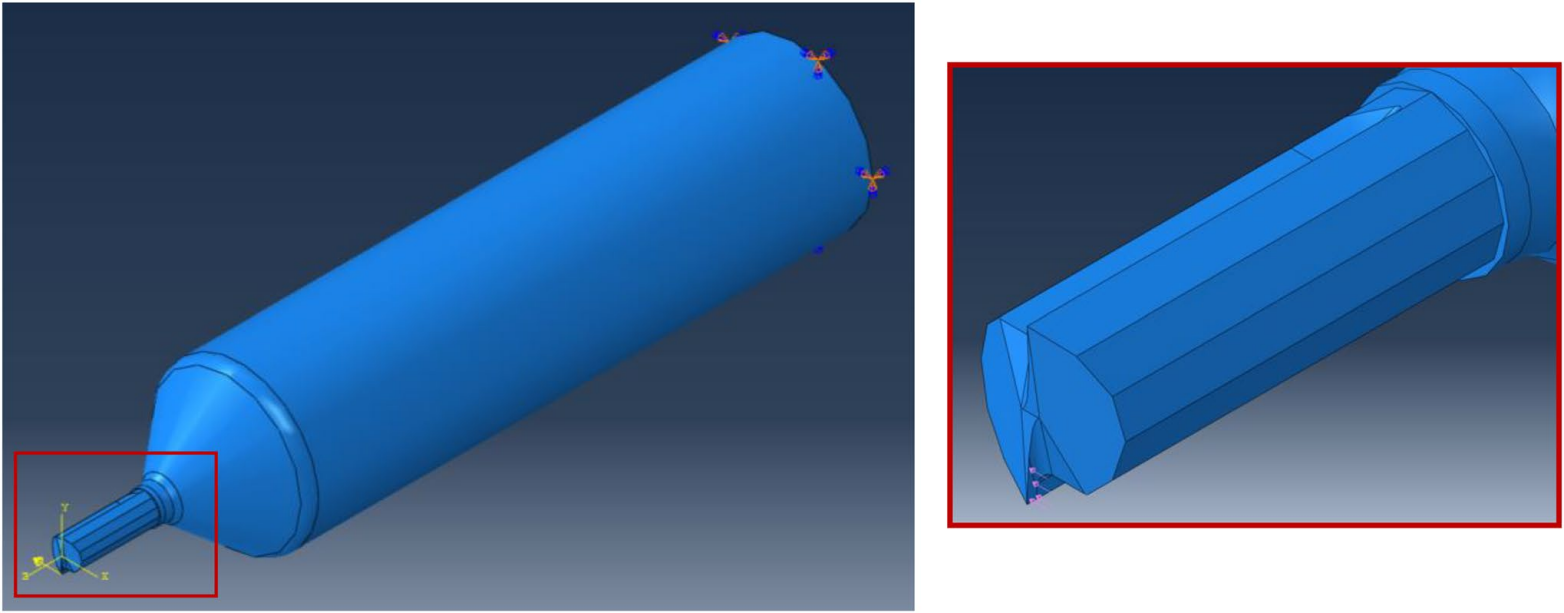
Load boundary conditions applied to tool, unclamped length, i.e., 18.5 mm length (yellow arrow is point load 3 N in *X* direction; orange and blue symbols indicate encastre boundary condition to simulate tool being clamped)

During the micro-milling process, the load condition on the cutting edge is complex and is a result of the infeed, crossfeed and thrust forces acting on the tool, which are directly related to the uncut chip thickness (UCT) [24], which in itself depends on the process parameters, material properties, and microstructure [25]. To simplify the cutting edge loading conditions for the analysis, the loading condition is applied as a distributed force along the surface area of the cutting tool where chip formation occurs, which is specified from the depth of cut and feed rate. This loading condition provides a realistic comparison with real micro-milling and allows for the estimation of the deflection and Von Mises stress at the cutting edge due to applied loads common to micro-milling of hard materials. During practical application, the clamped and unclamped lengths of tool in the spindle are 18.5 mm for both, so in this FEA model an encastre constraint is applied to the back face of the tool and only half the tool is modelled. The distributed loading condition is then applied to one of the cutting edges in *X* direction, as can be seen in Fig. 2.

**Fig. 3:**
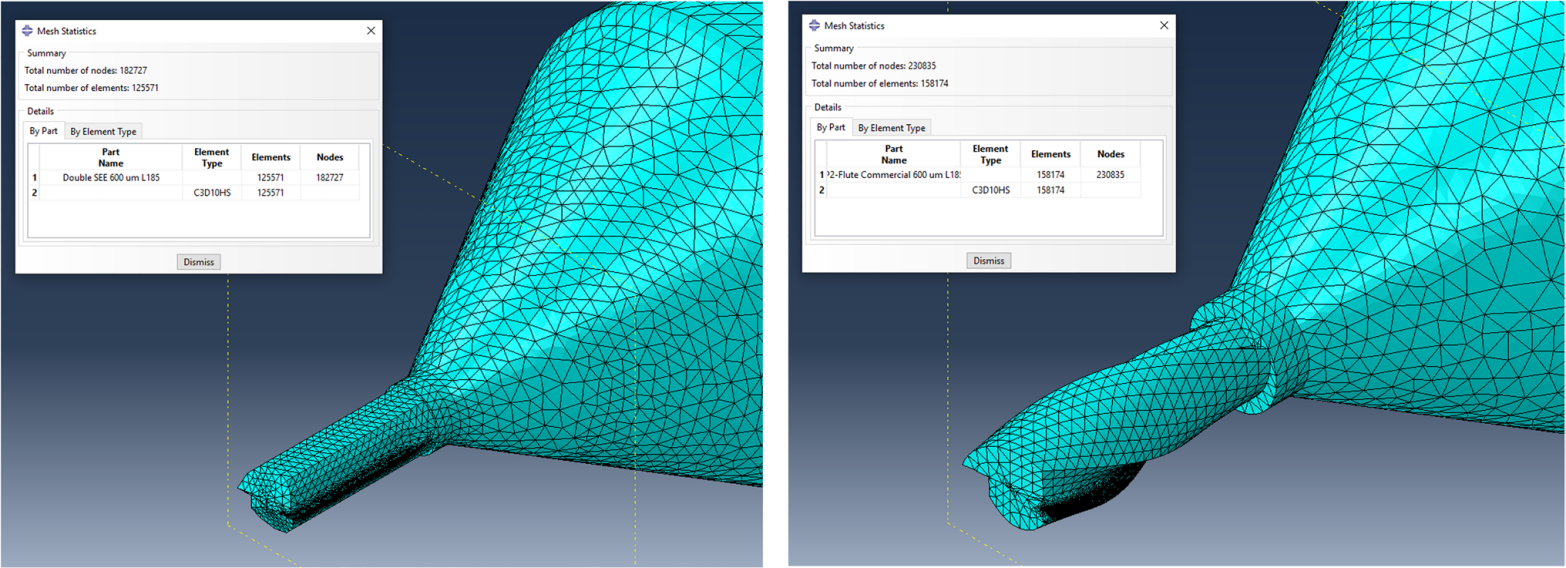
Mesh statistics and relative geometry of 600 µm diameter tool types, namely **a** DSEE and **b** 2-flute

The model definition is an Abaqus explicit 3D static stress/displacement analysis with 1 second step increment. Second-order elements provide higher accuracy in Abaqus/Standard than first-order elements for “smooth” problems that do not involve severe element distortions (i.e., point load). They capture stress concentrations more effectively and are better for modelling geometric features, i.e., they can model a curved surface with fewer elements. Finally, second-order elements are very effective in bending-dominated problems [26]. Hence, standard second-order10 node quadratic tetrahedron with improved surface stress visualisation (HS) elements was chosen, namely C3D10HS. This element type was chosen as they had good convergence rate with minimal shear or volumetric locking and were robust during finite deformation. The tool was partitioned into three sections to allow for better mesh refinement in the key sections. Approximate element size for 800 µm tool is as follows, tool section (i.e., shank up to cutting section) = 0.2, cutting section = 0.1, and cutting edge = 0.01. For the 600 µm tool, tool section = 0.15, cutting section = 0.075 and cutting edge = 0.01. A mesh convergence study was conducted until meshes yielded nearly identical results in relation to deflection analysis. The meshing and element information of each type of 600 µm diameter tool can be seen in Fig. 3.

Two types of tools were simulated using this model and the results of both the average and maximum deflection and Von Mises stress distributions were compared against each other. The designed DSEE tool was against a typical helical 2-flute tool design, for a range of tool diameters, namely 800 µm, 600 µm, 400 µm and 200 µm. The first set of simulations compares the DSEE against the 2-flute tools due to a distributed load of 3 N at the cutting edge. For the second set of simulations, we again compare the DSEE and 2-flute tools in relation to deflection and Von Mises stress, this time with a scaled distributed load with reduction in tool cutting diameter, simulating the differences of cutting force applied to each tool diameter during real application. Namely, 800 µm, 600 µm, 400 µm and 200 µm tools with 4 N, 3 N, 2 N, and 1 N distributed load, respectively. The material along the full length of the tools was WC, and the material properties were determined from Refs. [19, 27], as shown in Table 2.

**Table 2:** Material properties of WC tool for Abaqus CAE

Mechanical properties	
Density / (g · cm^–3^)	11.9
Thermal conductivity / (W · (m·K)^–1^)	60
Specific heat/(J·(Kg·K)^−1^)	250
Thermal expansion/K^–1^	5 × 10^−6^
Young’s modulus/GPa	800
Poison's ratio	0.22

**Fig. 4:**
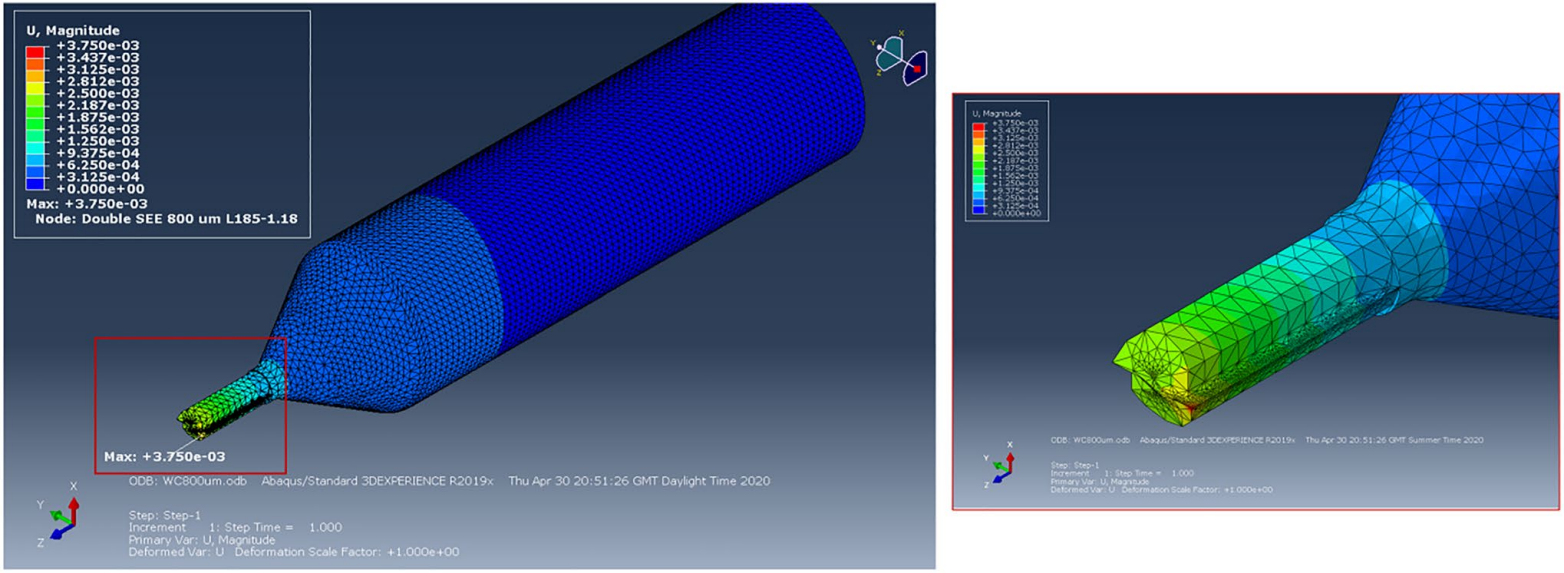
Abaqus contour plot of SEE 800 µm designed tool, detailing deflection due to point load

The results of each simulation are visualised using contour plots for deflection and Von Mises stress, taking the maximum values for the comparison between the designed tool and the common commercially available 2-flute design. The field output deflection gives an indication on the relative rigidity and stiffness of the tools, while Von Mises stress distribution identifies if the material at the cutting edge will yield or fracture under the applied distributed load. To more effectively evaluate the tool with real application, the results of the Von Mises stress are compared with the transverse rupture strength (TRS) of WC. TRS is a material property defined as the stress in a material before it yields, and therefore can be used to compare against the Von Mises principal stress to determine if tool breakage will occur during the simulation. The TRS for WC tools throughout this work is considered to be 3.2 GPa. An example of the contour plot of deflection is shown in Fig. 4.

### FEA results

**Fig. 5:**
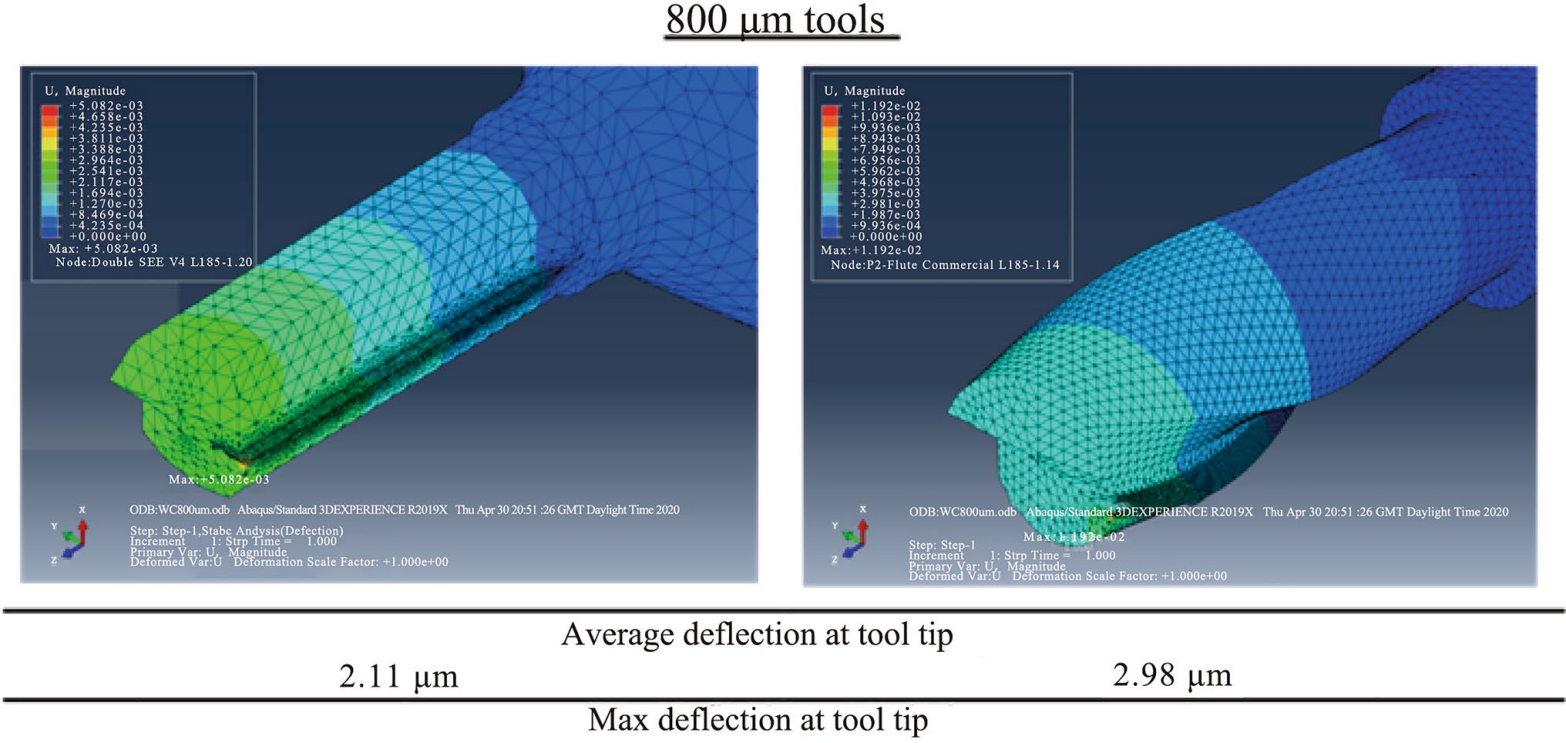
Typical Abaqus CAE FEA simulation results for both DSEE and 2-flute design 800 µm tools in regards to the average and maximum deflection

**Fig. 6:**
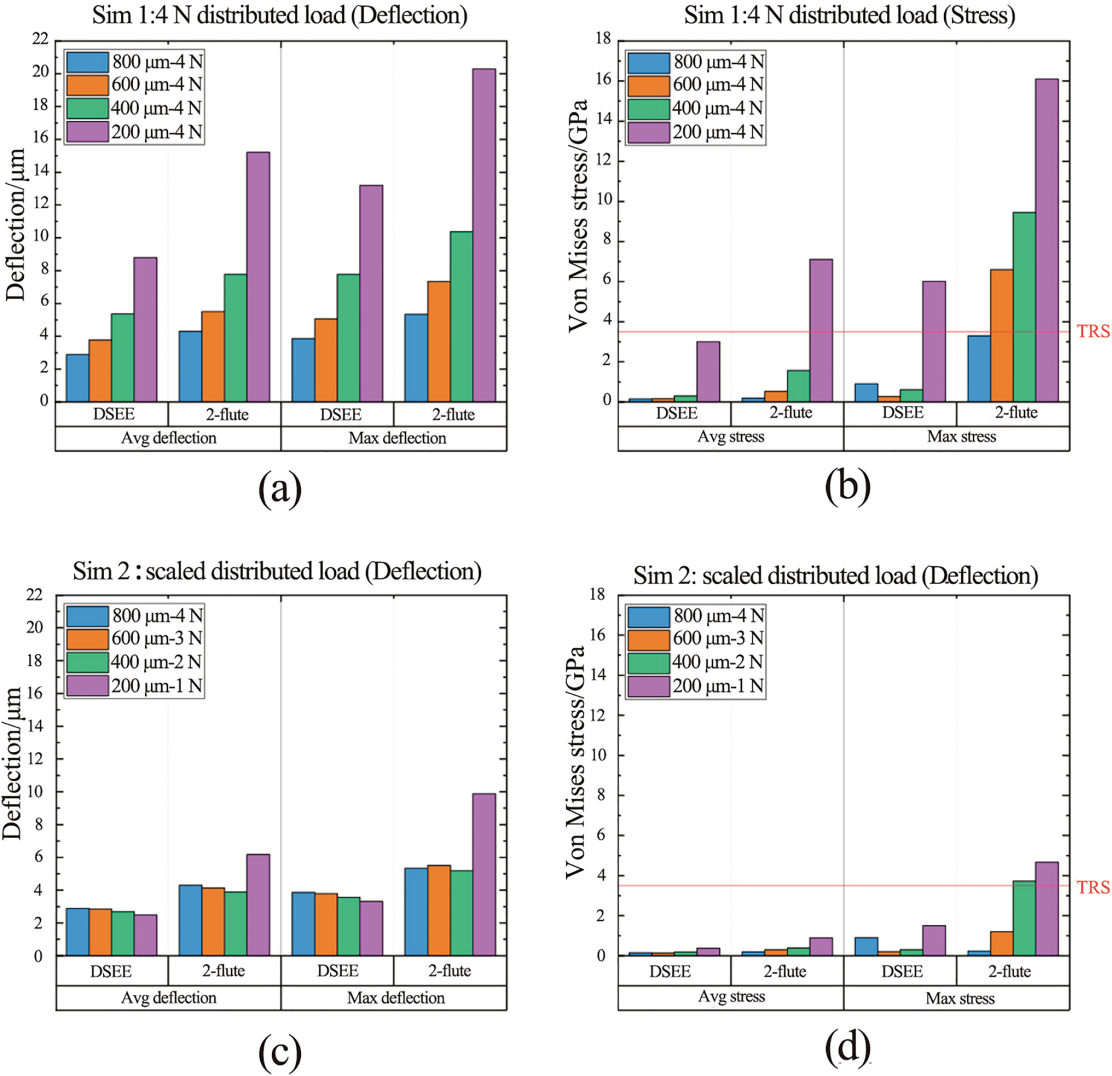
Results of simulation set 1 for both types of tool designs **a** in relation to deflection and **b** Von Mises stress, and results of simulation set 2 for both types of tool designs **c** in relation to deflection and **d** Von Mises stress

Figure 5 shows typical Abaqus CAE FEA simulation results for both DSEE and 2-flute design 800 µm tools. The results of the first set of simulations on both tool designs, and for average and max deflection, can be seen in Figs. 6a, b. In regards to deflection, the DSEE 800 µm, 600 µm, 400 µm and 200 µm tools have average deflections of 2.89 µm, 3.78 µm, 5.36 µm and 8.80 µm, with the maximum deflections of 3.85 µm, 5.06 µm, 7.78 µm and 13.20 µm, respectively. While the 2-flute 800 µm, 600 µm, 400 µm and 200 µm tools have average deflections of 4.30 µm, 5.51 µm, 57.78 µm and 13.20 µm, with the maximum deflections of 5.34 µm, 7.34 µm, 10.37 µm and 20.29 µm. The results show that the DSEE tool has a reduction of deflection of 20% for 800 µm tool, 25% for 600 µm tool, 25% for the 400 µm tool and 25% for the 200 µm tool, in comparison to the 2-flute tool. Both average and the maximum deflections of the tool tip are significantly reduced for the DSEE tools in comparison to 2-flute design through the range of diameters, showing good scalability and the potential for even smaller diameter tools using this design. The average deflection values of less than 5 µm give good indication on the relative rigidity and stiffness of this tool design, which satisfies the objectives of the design specification. In relation to Von Mises stress, the DSEE 800 µm, 600 µm, 400 µm and 200 µm tools have average Von Mises stress of 0.15 GPa, 0.16 GPa, 0.30 GPa and 3.0 GPa, with the maximum Von Mises stress of 0.901 GPa, 0.27 GPa, 0.61 GPa and 6.01 GPa, respectively. While the 2-flute 800 µm, 600 µm, 400 µm and 200 µm tools have average Von Mises stress of 0.19 GPa, 0.53 GPa, 1.57 GPa and 7.10 GPa, with the maximum Von Mises stress of 3.30 GPa, 6.59 GPa, 9.45 GPa and 16.10 GPa, respectively. The results again show that the DSEE design tools show better stress distribution and lower maximum stresses at the location of the distributed load, in comparison to the 2-flute commercial design over all tool diameters. The maximum stress at the cutting edge was reduced by 95% for 800 µm, 92% for 600 µm, 76% for 400 µm and 81% for 200 µm tool. These results show that high strength can be imparted into micro-milling tools by increasing the volume around the cutting edge and peripheral cutting edge through optimized geometric design of the tool.

The results of the second set of simulation results, i.e., investigation of scaled cutting forces, are presented in Figs. 6c, d. The purpose of reducing the applied distributed load with tool diameter is to better reflect the real cutting forces for these tool diameters during actual micro-milling and to compare the relative stiffness, rigidity and strength between the two designs over the range of diameters. Again in regards to deflection, the DSEE 800 µm, 600 µm, 400 µm and 200 µm tools have average deflections of 2.89 µm, 2.85 µm, 2.68 µm and 2.48 µm, for distributed loads of 4 N, 3 N, 2 N and 1 N, respectively. In comparison, the 2-flute 800 µm, 600 µm, 400 µm and 200 µm tools have average deflections of 4.30 µm, 4.13 µm, 3.89 µm and 6.17 µm for distributed loads of 4 N, 3 N, 2 N and 1 N. The results clearly show a significant reduction in average tool deflection for the DSEE tools in comparison to 2-flute tools, namely 33% reduction for 800 µm, 31% for 600 µm, 31% for 400 µm and 60% for 200 µm tools. Similarly, in relation to stress, the DSEE 800 µm, 600 µm, 400 µm and 200 µm tools have average Von Mises stress of 0.15 GPa, 0.14 GPa, 0.18 GPa and 0.38 GPa for the distributed loads of 4 N, 3 N, 2 N and 1 N, respectively. While the 2-flute 800 µm, 600 µm, 400 µm and 200 µm tools have average Von Mises stress of 0.19 GPa, 0.20 GPa, 0.30 GPa and 1.50 GPa for the same distributed load case. This leads to reduction in Von Mises average stress for the DSEE tool design of 21% for 800 µm, 30% for 600 µm, 40% for 400 µm and 75% for 200 µm tools. Comparing the results of the DSEE tools with the 3.2 GPa TRS value for WC material in Figs. 6b, d, none of the tools would not chip or break significantly due to the applied loads. In comparison, all 2-flute design tool diameters would experience significant chipping and tool breakage as the Von Mises stress values are significantly higher than the TRS value for WC. Furthermore, the DSEE 800 µm, 600 µm and 400 µm tools show excellent strength, even at the relatively high loads applied, indicating this tool design is suitable for micro-milling of very hard materials. Overall, these results clearly show that the DSEE tools have better stress distribution than the 2-flute tools due to the increased volume behind the cutting edge and larger cross sectional area, imparting higher strength to the cutting edge. It also indicates this tool design is robust across all diameters and that it has satisfied the developed tool criterion for micro-milling of very hard materials.

## Experimental validations

**Fig. 7:**
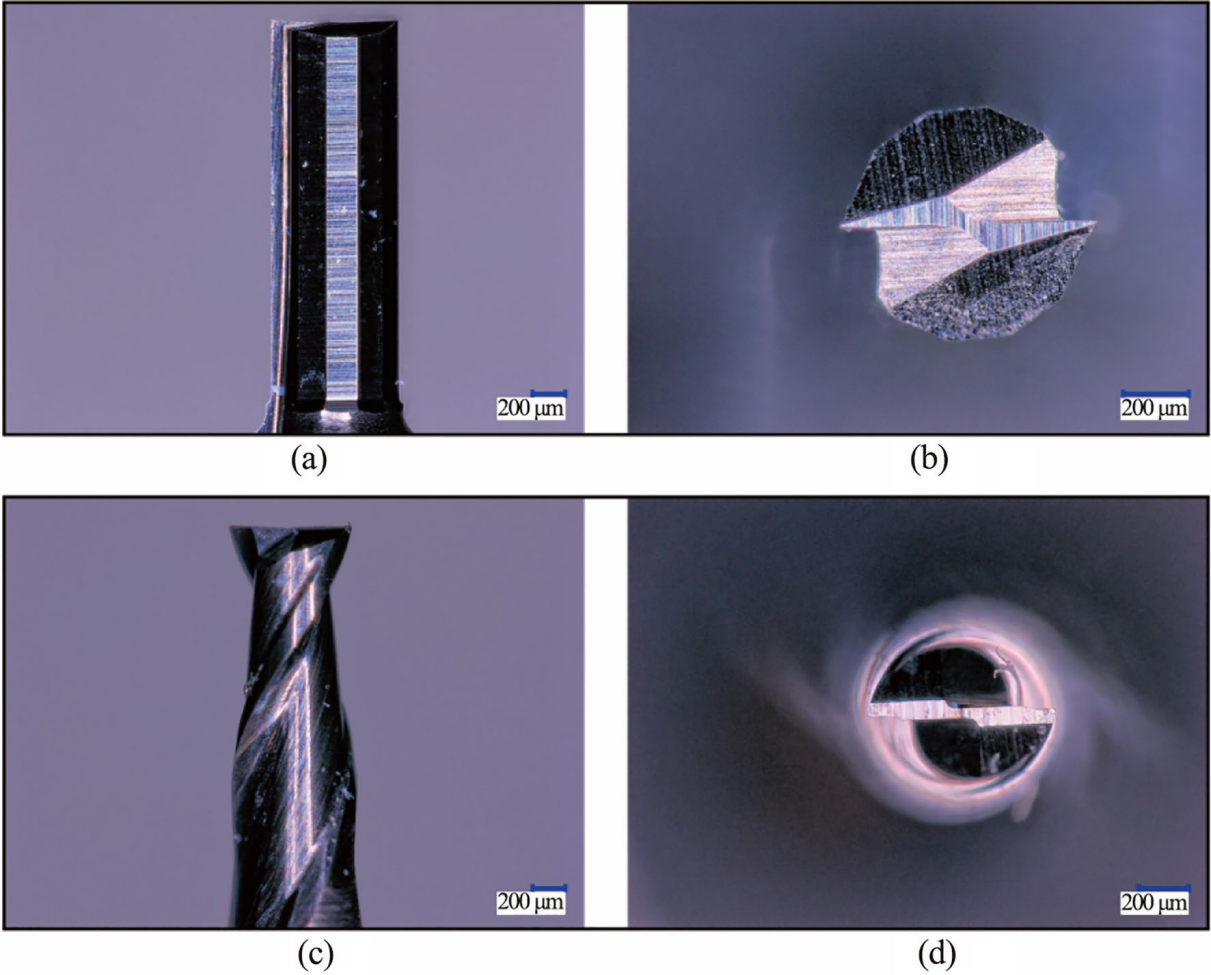
Images of the DSEE and conventional 2-flute tool **a** side view DSEE tool, **b** top view DSEE tool, **c** side view 2-flute tool and **d** top view 2-flute tool

With the results of the FEA simulation, the DSEE tools were then fabricated for diameters 800 µm and 600 µm from fine grain WC. Images of both the designed DSEE tool and conventional 2-flute tools can be seen in Fig. 7. A fractional parametric experimental study was then carried out on the fabricated tools to compare the developed tool design to commercially available 2-flute tools. The purpose of these experiments was to evaluate the geometrical tool design of the DSEE micro-milling tools in comparison to such 2-flute tools of the same tool material, specifically for micro-milling of very hard DTM materials. Both tool types were subjected to the same experiment method and process parameters in order to examine and compare the results between both, and to determine the effectiveness of the DSEE design. The significant process outputs were cutting force, surface roughness, burr formation, chip size and tool wear. The results were then used to evaluate both tool types over both diameters and workpiece materials.

### Experimental method

The examined process outputs during experiments were cutting force, surface quality, in regard to surface roughness and burr formation, chip size and tool wear. The process input parameters were tool design, workpiece material, spindle speed and feed rate. The workpiece materials were ISO 5832-4 cobalt-chromium-molybdenum (CoCrMo) alloy and ISO 5832-3 titanium (Ti6Al4V) alloy, and were precision ground flat and to surface roughness of around 100 nm in surface roughness (*S*_a_) prior to experiments. Tool materials for both the developed tool and high quality commercial tool were high grade WC with very similar dimensions. Both were fabricated by Louis Belet Swiss Cutting Tools and were uncoated. The control 2-flute tool was “Micro end mill Z2”. The parameters for each experiment were depth of cut, which was 50 µm and 35 µm for the 800 µm and 600 µm diameter tools, and length of cut 2 mm and 1.5 mm, respectively. The experiments were full immersion up slot micro-milling, ran under dry machining conditions. A Taguchi *L*36 [22, 23] orthogonal array, 2 levels for 2 factors and 3 levels for 2 factors, is implemented as the fractional factorial design of experiments, which allows for the examination of pairs of input parameters to determine which factors most affect process outputs with a minimum amount of tools necessary. Blocks are used to assign portion of the experiment that are more homogenous in the experiment set, by comparing conditions of interest within each block. Therefore, spindle speed was blocked while feed rate was varied as it had the most significant impact on process outputs. The experiments were also run in a randomised order. The machining process parameters for each tool diameter are listed in Table 3, while Table 4 is a typical experiment run for DSEE 800 µm tool, detailing how blocks are implemented.

**Table 3:** Machining parameters for both tool diameters

Factors				Levels		
				1	2	3
Tool diameter—800 µm						
1	Tool design			DSEE	2-flute	–
2	WP material			CoCrMo	Ti6Al4V	–
3	Spindle speed, *N*/ (r · min^–1^)			20 000	25 000	30 000
4	Feed rate, *V*_F_ / (mm · min^–1^)			8, 16, 24	10, 20, 30	12, 24, 36
	Feed per tooth, *F*_z_ /μm			0.2, 0.4, 0.6		
Tool diameter—600 µm						
1	Tool design			DSEE	2-flute	–
2	WP material			CoCrMo	Ti6Al4V	–
3	Spindle speed, *N*/ (r · min^–1^)			30 000	35 000	40 000
4	Feed rate, *V*_f_ / (mm · min^–1^)			12, 24, 36	14, 28, 42	16, 32, 48
	Feed per tooth, *F*_z_ /μm			0.2, 0.4, 0.6

**Table 4:** Experimental run conditions for both tool designs for 800 µm diameter tools

		Exp No.	Tool design	WP material	Spindle speed *N* /(r · min^−1^)	Feed rate *V*_f_ /(mm · min^−1^)
DSEE	Block—Tool 1	1	1	CoCrMo	20 000	8
		2	1	CoCrMo	20 000	16
		3	1	CoCrMo	20 000	24
	Block—Tool 2	4	1	CoCrMo	25 000	10
		5	1	CoCrMo	25 000	20
		6	1	CoCrMo	25 000	30
	Block—Tool 3	7	1	CoCrMo	30 000	12
		8	1	CoCrMo	30 000	24
		9	1	CoCrMo	30 000	36
	Block—Tool 4	1	1	Ti6Al4V	20 000	8
		2	1	Ti6Al4V	20 000	16
		3	1	Ti6Al4V	20 000	24
	Block—Tool 5	4	1	Ti6Al4V	25 000	10
		5	1	Ti6Al4V	25 000	20
		6	1	Ti6Al4V	25 000	30
	Block—Tool 6	7	1	Ti6Al4V	30 000	12
		8	1	Ti6Al4V	30 000	24
		9	1	Ti6Al4V	30 000	36
2-flute	Block—Tool 1	1	2	CoCrMo	20 000	8
		2	2	CoCrMo	20 000	16
		3	2	CoCrMo	20 000	24
	Block—Tool 2	4	2	CoCrMo	25 000	10
		5	2	CoCrMo	25 000	20
		6	2	CoCrMo	25 000	30
	Block—Tool 3	7	2	CoCrMo	30 000	12
		8	2	CoCrMo	30 000	24
		9	2	CoCrMo	30 000	36
	Block—Tool 4	1	2	Ti6Al4V	20 000	8
		2	2	Ti6Al4V	20 000	16
		3	2	Ti6Al4V	20 000	24
	Block—Tool 5	4	2	Ti6Al4V	25 000	10
		5	2	Ti6Al4V	25 000	20
		6	2	Ti6Al4V	25 000	30
	Block—Tool 6	7	2	Ti6Al4V	30 000	12
		8	2	Ti6Al4V	30 000	24
		9	2	Ti6Al4V	30 000	36

To characterise tool wear more accurately, a tool wear criterion was developed. This criterion characterised each tool regarding type of tool wear occurring, on both flutes/edges, how much wear occurred, the overall condition of the tool, a recommendation on when to change the tool, and finally information on tool runout. The criterion considers two methods for the determination of tool condition. The first is average tool wear between both flutes on the rake and flank faces, while the second is reduction in tool diameter, to fully encapsulate the state of the tool. For the first method, a tool wear formula was developed from the analysis of the results of cutting force and surface roughness, to determine how much wear occurred on each face and each flute. The formulae for both are below. The tool condition parameter (*T*_P_) in Eq. (1) was determined from analysis of tool wear during experiments, i.e., 0.05 for “New”, 0.1 for “Change Soon”, 0.2 for “Change Now” and > 0.2 for “Complete Failure”. A parameter of 0.025 can also be used for “Chipping”. The calculation and results of the wear area formula (*A*_W_) give a value for the area of tool wear which occurred, as seen in Tables 5, 6. The 2-flute tools have a small concave section of material missing when viewed from above, i.e., along the flank face, which is roughly 400 µm^2^ and 300 µm^2^ for 800 µm and 600 µm diameter tools, respectively. Therefore this area was removed from calculation of flank wear area.

1 $$A_{\text{W}} = T_{\text{P}} \times A_{\text{CE}},$$

2 $$A_{\text{CE}} = D_{\text{C}} \times L_{\text{C}},$$

where *A*_CE_ is cutting edge area.

**Table 5:** Rake and flank face tool wear characterisation and tool conditions

Characterisation	Condition	Wear area formula	800 µm tool /µm^2^	600 µm tool /µm^2^
New	Good	0.05*A*_CE_	350	187.5
Minor wear	Change soon	0.1*A*_CE_	700	375
Major wear	Change now	0.2*A*_CE_	1 400	750
Complete failure	Too late	> 0.2*A*_CE_	> 1 400	> 750
Chipping		> 0.025*A*_CE_

**Table 6:** *A*_CE_ characterisation and values

	*D*_C_ × *L*_C_ /µm^2^	*A*_CE_ /µm^2^
DSEE 800 µm	50.0 × 140	7 000
DSEE 600 µm	37.5 × 100	3 750
2-flute 800 µm	50.0 × 140	7 000
2-flute 600 µm	37.5 × 100	3 750

**Fig. 8:**
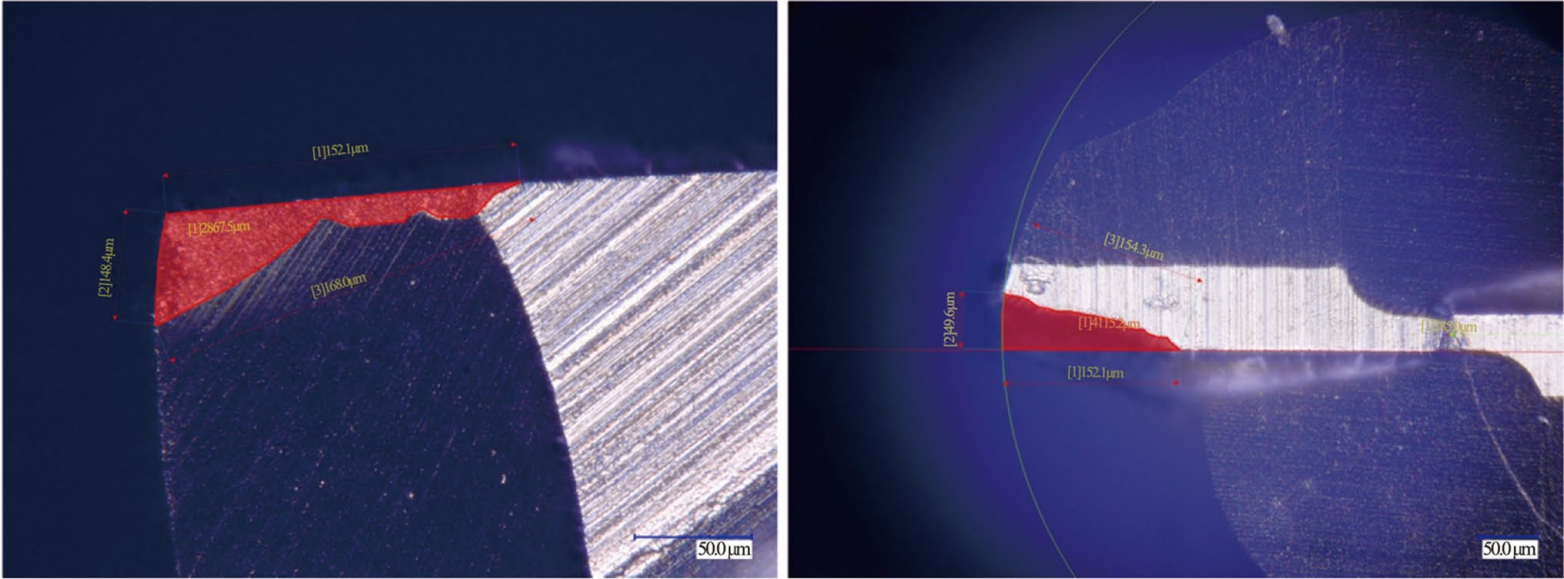
Method of wear area measurement of **a** tool rake and **b** flank faces

The second method for characterising tool wear only considers the reduction in outer cutting diameter of the tool. The formula for characterising reduced diameter (*D*_r_) uses different values for *T*_P_ and is the original diameter (*D*_o_) multiplied by *T*_P_, as shown in Eq. (3). The results of reduced diameter again give lower bounds for how much wear is acceptable before action must be taken. The equation below shows the reduced diameter calculation, and Table 7 shows allowable *D*_r_ for each tool condition. It is important to take both methods into account to fully characterise tool wear and determine tool condition. The first method will determine flank and rake face condition as well as identify tool runout, which will significantly impact surface quality and burr formation, while the second method will determine overall cutting diameter reduction of the tool, which will affect the geometrical tolerance of the machined feature. Figure 8 shows how the wear area is measured for rake face, and for flank face and reduced diameter, using the Keyence optical profiler.

3 $$D_{{\text{r}}} = { }D_{{\text{o}}} \times T_{\text{P}}{.}$$

**Table 7:** Reduced diameter tool wear characterisation and tool conditions

Characterisation	Condition	Reduced diameter	800 µm tool /µm	600 µm tool /µm
New	Good	0.05 *D*	760	570
Minor wear	Change soon	0.075 *D*	740	555
Major wear	Change now	0.1 *D*	720	540
Complete failure	Too late	> 0.1 *D*	< 720	< 540

### Experimental setup

Experiments were conducted on an ultra-precision 4 axis machining centre with CNC in a temperature-controlled lab at (20 ± 1) °C. A Kistler dynamometer Type 9119AA2 was mounted on a backing plate and mounted on the work holding spindle of the machine to measure three component cutting forces, *F*_*x*_, *F*_*y*_ and *F*_*z*_, during the micro-milling experiments. This dynamometer has very low threshold of lower than 0.002 N, low crosstalk between channels of ≤ ± 2%, high natural frequency and very high sensitivity which allows measurement of extremely small force.The sensitivity of the dynamometer is 26 pC/N in the *F*_*x*_ and *F*_*z*_ directions and 13 pC/N in the *F*_*y*_ direction, and the natural frequency of the device is *f*_*n*_ (*x*) ≈ 4.3 kHz, *f*_*n*_ (*y*) ≈ 4.6 kHz and *f*_*n*_ (*z*) ≈ 4.4 kHz. A sampling rate of 25 kHz was used which was far higher than the natural frequency of the dynamometer. The dynamometer was aligned such that the crossfeed cutting force was in the *F*_*x*_ direction, infeed cutting force in the *F*_*y*_ direction and thrust force in the *F*_*z*_ direction. The workpiece materials, in wrought cylindrical bar form, with dimensions of 30 mm diameter and 20 mm height, was clamped into a holding fixture which in turn was bolted to the dynamometer. The dynamometer was connected to a Kistler Lab Amp Type 5167AX1, which was both a charge amplifier for multi-component force measurements, as well as a data acquisition device. The signal data were then analysed and processed on a host computer using the Kistler DynoWare software.

**Fig. 9:**
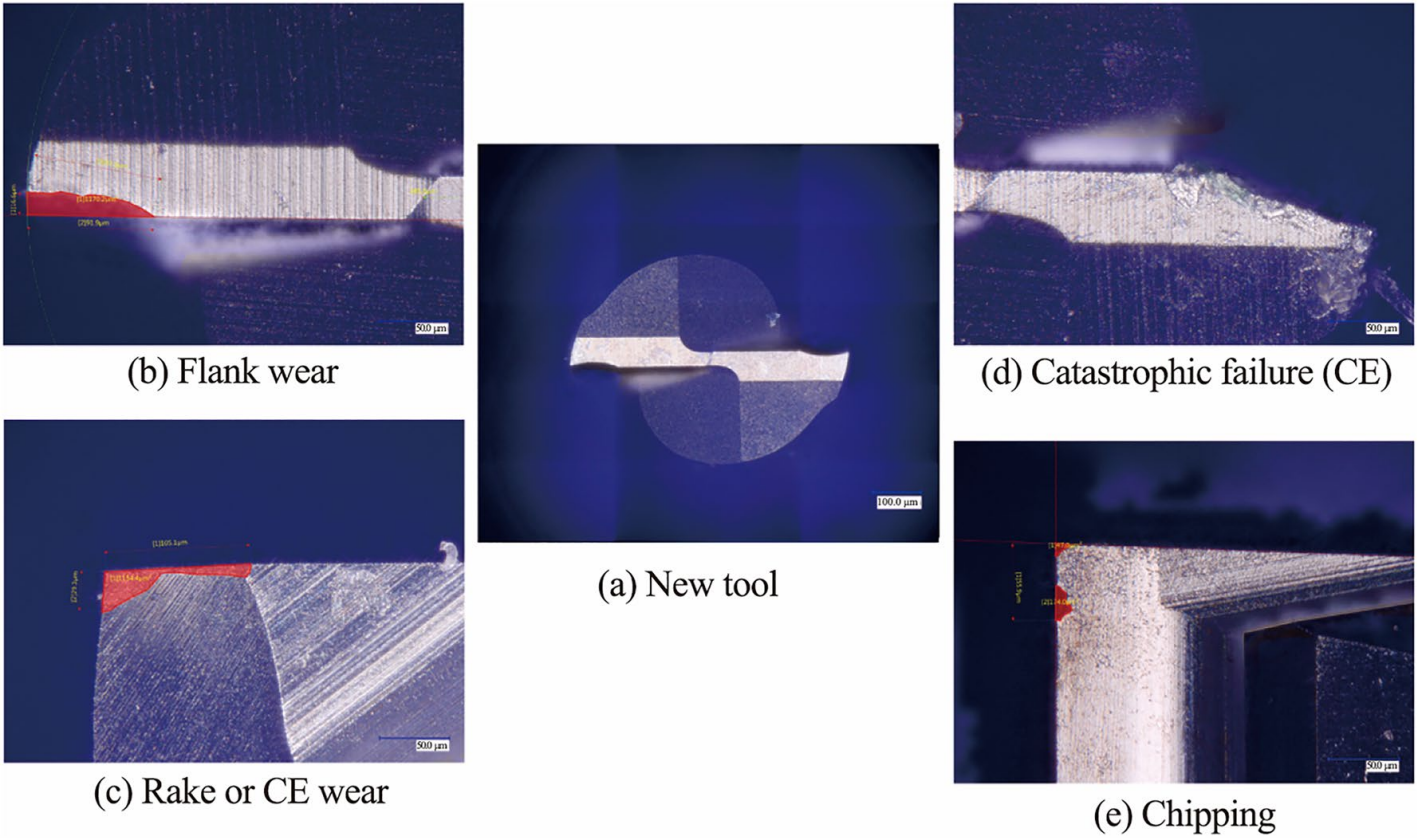
Identified modes of tool wear during micro-milling experiments

Each of the experiment blocks was prescribed a new tool (Exps 1, 4 and 7), and measured after each experiment for analysis of tool wear on a Keyence VHX-5000 optical microscope. Measurement of tool wear involved identifying modes of tool wear that occurred, as seen in Fig. 9, conducting surface area measurement of the flank and rake faces using area measurement function on the Keyence software, and finally developing a tool wear criterion to determine tool condition post machining. This method only considers 2D data, which determine tool wear as a result of missing cutting edge or severely degraded tool surface. However, a more robust 3D scanned mesh, such as developed by Petrò and Moroni [28], could be implemented with the developed tool wear criterion. Surface topography measurements were then carried out on the machined surface using a Bruker NPFlex 3D surface metrology system to determine surface roughness in relation to arithmetical mean height. A Gaussian Regression filter, data masking and tilt removal were then applied. Masks were used to determine *S*_a_ at the start, end and over the entire machined surface of the slot. Burr formation was qualitatively analysed and characterised optically, using the Keyence microscope at ×500 magnification at key areas along the slot. Finally, cutting chips were collected and observed under the microscope at high magnification of ×2 000 to determine chip size and shape. Ten measurements of both length and width dimensions were taken for each chip, from which the average chip dimension was plotted with the standard deviation.

### Experimental results

#### Cutting force

**Fig. 10:**
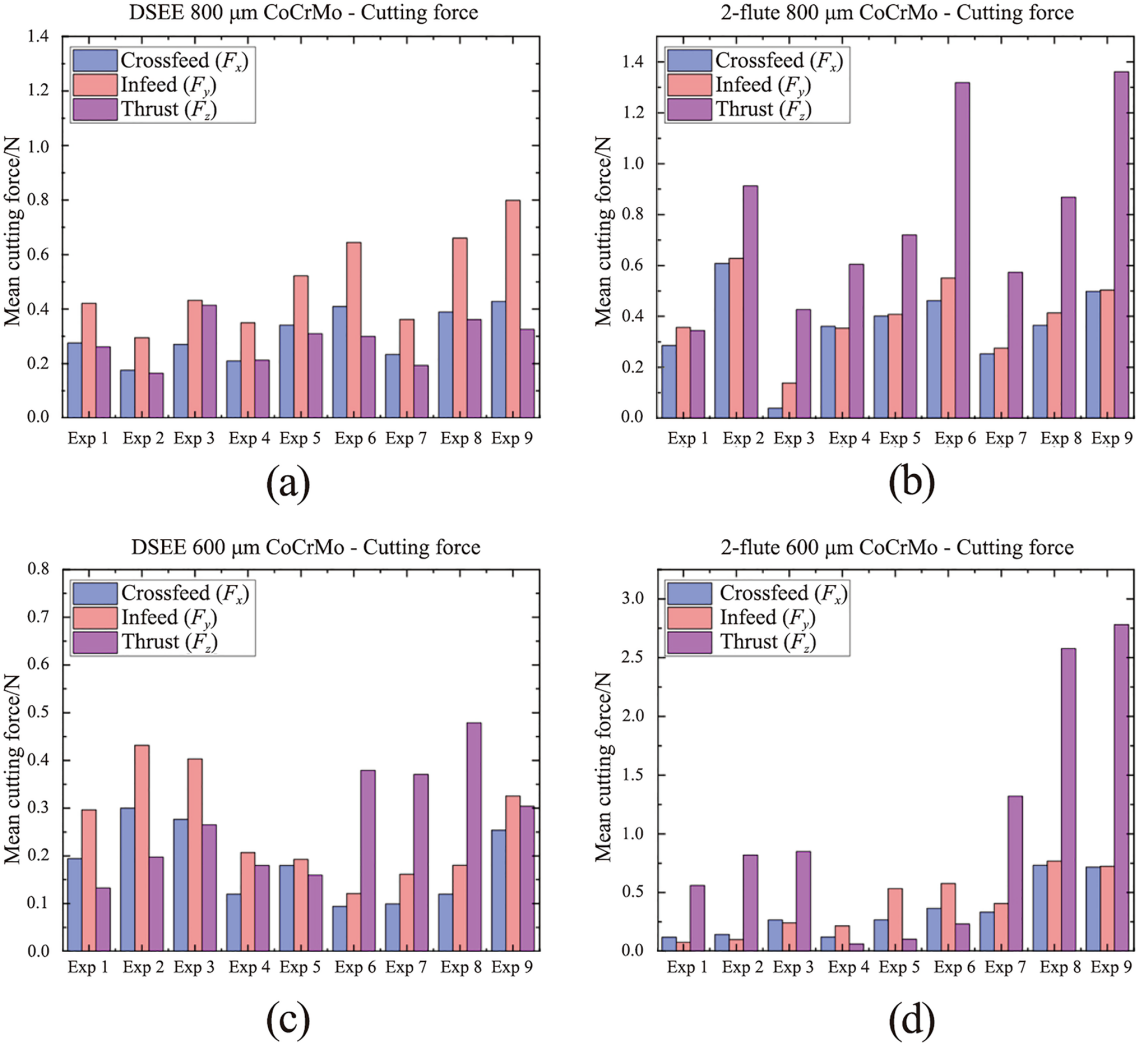
Mean cutting force for each experiment on CoCrMo workpiece material, comparing **a** DSEE 800 µm versus **b** 2-flute 800 µm, and **c** DSEE 600 µm versus **d** 2-flute 600 µm

**Fig. 11:**
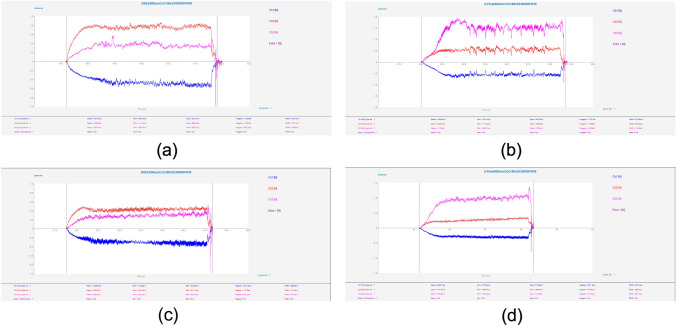
Cutting force data on CoCrMo workpieces for both tool types and tool diameters with the same process parameters of *N* = 30 000 r/min and *V*_f_ = 36 mm/min. Exp 9 for **a** DSEE 800 µm versus **b** 2-flute 800 µm, and Exp 3 for **c** DSEE 600 µm vesus **d** 2-flute 600 µm

**Fig. 12:**
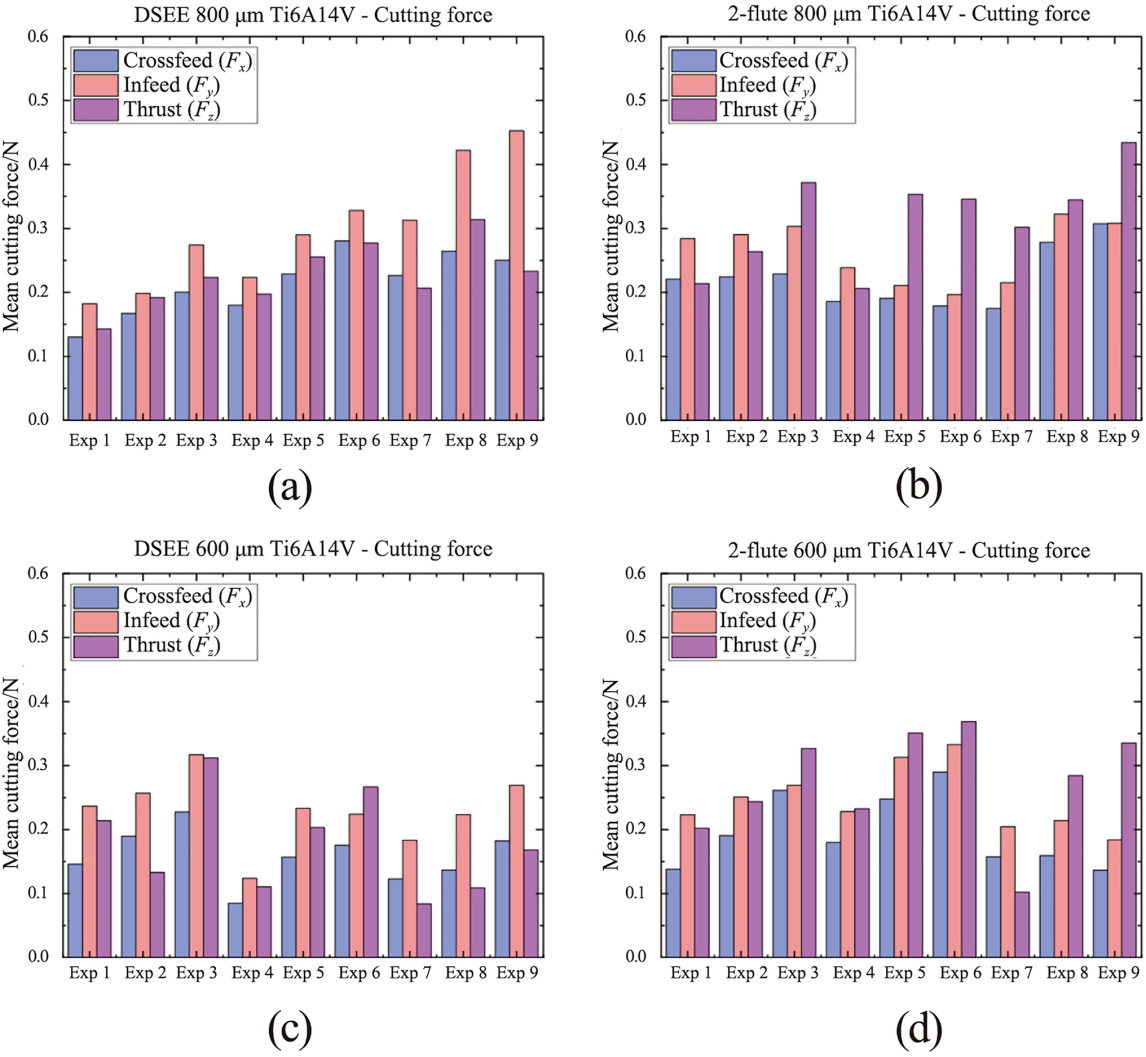
Mean cutting force for each experiment on Ti6Al4V workpiece material, comparing **a** DSEE 800 µm versus **b** 2-flute 800 µm, and **c** DSEE 600 µm versus **d** 2-flute 600 µm

Results of mean cutting forces in the three component directions throughout each experiment for the CoCrMo workpiece material for both diameters and tool types are displayed in Fig. 10. The DSEE tool shows overall much lower cutting forces than the 2-flute tools for this workpiece material (see Fig. 11). The mean crossfeed (*F*_*x*_) cutting force, in blue, is lower for DSEE tools, while infeed (*F*_*y*_) in red, is similar for both tool types. However, thrust force (*F*_*z*_) in pink, is significantly lower for DSEE tool in comparison to 2-flute tool and these results are consistent over both tool diameters. It also must be noted that cutting force was affected by significant tool wear that occurred during 2-flute 800 µm Exps 3 and 6, while complete tool breakage occurred during Exps 8 and 9. The results for mean cutting force for the Ti6Al4V workpiece material are shown in Fig. 12. The results of the Ti6Al4V workpiece material generally follow the overall trend of the CoCrMo workpiece material results. Both tool types display similar crossfeed cutting force, slightly higher infeed cutting force for DSEE and lower thrust cutting force for DSEE tool than the 2-flute tool. Again, complete tool breakage also occurred during Exp 9 of 2-flute 800 µm and Exp 3 of 2-flute 800 µm. The results for mean cutting force for both workpiece materials show that cutting force increases with feed rate, per block of spindle speeds. Higher spindle speed blocks with higher feed rate also result in higher cutting forces, i.e., comparing Exps 1–3, 4–6, and 7–9, across both tool diameters, tool types and workpiece materials.

**Fig. 13:**
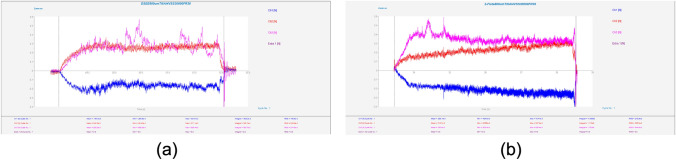
Cutting force data on Ti6Al4V workpieces for both tool types of diameter 800 µm with the same process parameters of *N* = 30 000 r/min and *V*_f_ = 36 mm/min. Exp 9 for **a** DSEE 800 µm versus **b** 2-flute 800 µm

Typical cutting force data for equivalent experiments comparing the DSEE and 2-flute 800 µm tools are presented in Figs. 11a, b for CoCrMo workpiece material. For the DSEE 800 µm tool, crossfeed cutting force is 0.5 N; infeed is 0.8 N and axial is 0.4 N. The cutting force signal is also very stable. In comparison, for the 800 µm 2-flute tool, the crossfeed cutting force is 0.5 N; infeed is 0.5 N and axial is 1.3 N. Also, the cutting force signal is inherently unstable during machining experiments. The infeed cutting force is slightly lower for the 2-flute tool compared to the DSEE tool due to the straight edge design of the DSEE tool, which directs chip flow radially inwards towards the centre of rotation. Similarly, the thrust force is far lower for the DSEE tool, again due to straight edge design. Whereas chip flow is directed up and away from working zone on the 2-flute tool, leading to significantly higher forces in the thrust direction. These results are consistent with the 600 µm tools, as seen in Figs. 11c, d. For the DSEE 600 µm tool, crossfeed cutting force is 0.27 N; infeed is 0.4 N and thrust is 0.27 N. In comparison, for the 600 µm 2-flute tool, the crossfeed cutting force is 0.25 N; infeed is 0.25 N and axial is 0.85 N. Again, typical cutting force data for equivalent experiments comparing the DSEE and 2-flute 800 µm tools are presented in Fig. 11 for Ti6Al4V. For the DSEE 800 µm tool, crossfeed cutting force is 0.2 N; infeed is 0.25 N and axial is 0.25 N. In comparison, for the 800 µm 2-flute tool, the crossfeed cutting force is 0.2 N; infeed is 0.2 N and axial is 0.35 N. The cutting force signal during machining of Ti6Al4V workpiece material is relatively less stable than for CoCrMo for both tool types, as seen in Fig. 13. However, cutting forces are much lower for machining of Ti6Al4V with the 800 µm tool diameters for both tool types, and relatively similar for 600 µm tool diameters.

#### Surface roughness

**Fig. 14:**
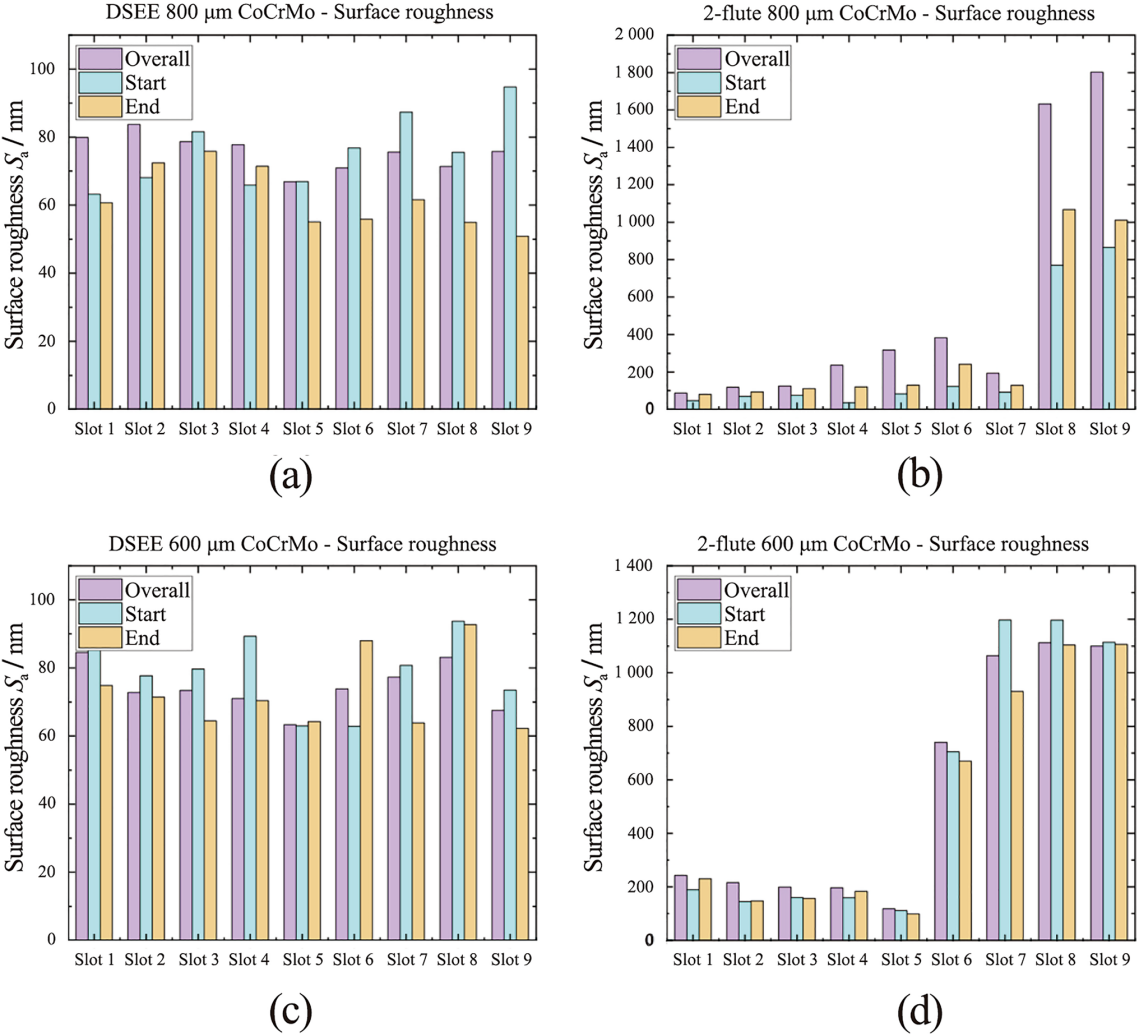
*S*_a_ values for each experiment slot on CoCrMo workpiece material, measuring in three locations (overall, start and end), comparing **a** DSEE 800 µm versus **b** 2-flute 800 µm, and **c** DSEE 600 µm versus **d** 2-flute 600 µm

**Fig. 15:**
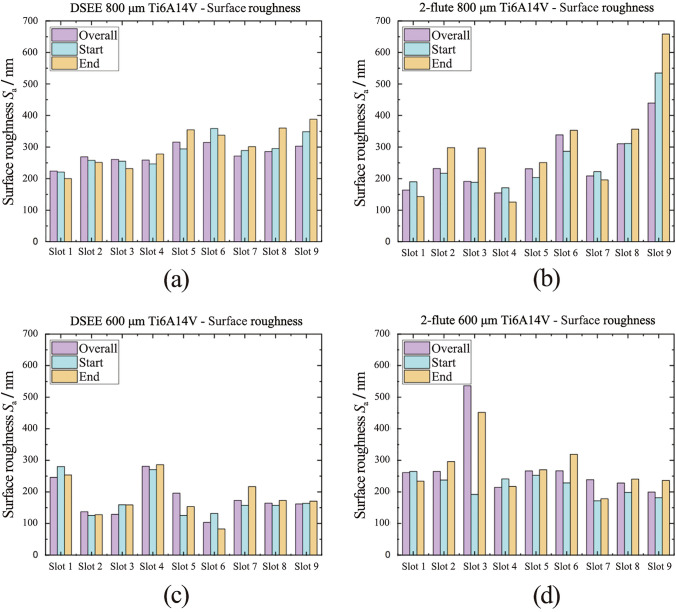
*S*_a_ value for each experiment slot on Ti6Al4V workpiece material, measuring in three locations (overall, start and end), comparing **a** DSEE 800 µm versus **b** 2-flute 800 µm, and **c** DSEE 600 µm versus **d** 2-flute 600 µm

The results of *S*_a_ analysis are presented below in Fig. 14 for workpiece material CoCrMo for both tool types and diameters, and Fig. 15 for workpiece material Ti6Al4V. Very similar overall *S*_a_ (purple) values were measured across all DSEE 800 µm experiments for CoCrMo. Considering each tool was used for 3 experiments, i.e., tool 1 for Exps 1–3, tool 2 for Exps 4–6, etc., only minor increases in surface roughness were measured after each tool use. This indicates that overall, only very little tool wear occurred and the tool remained robust without edge chipping throughout each experiment block. Surface roughness increases at start (blue) through Exps 1–3, Exps 4–6 and Exps 7–9, as less stable machining occurs at the start of the slot where cutting force ramps up. However, lower surface roughness at the end of the slots (yellow) than at start indicates very minor tool wear reduced the tool edge radius, which is known to help improve surface roughness values [29]. The other reason for lower surface roughness values at the end of the slots is due to more stable machining conditions at the end of the slot, as identified in cutting force data. The results are very similar for DSEE 600 µm tool on the same workpiece material. Feed rate had only minor effect on the surface roughness, with the lowest *S*_a_ values occurring in Exp 5 for both tool sizes, namely 25 000 r/min spindle speed and 20 mm/min feed rate for the DSEE 800 µm, and 35 000 r/min spindle speed and 28 mm/min feed rate for the DSEE 600 µm tool. Increasing feed rate within each spindle speed block only slightly increased surface roughness, as did increasing spindle speed through each block. Overall, micro-milling experiments with the DSEE tools produced very low surface roughness values, with *S*_a_ < 0.1 µm across all experiments on the CoCrMo workpiece. The results from the Ti6Al4V workpiece material follow the same trends for both DSEE tool diameters, as seen in Figs. 15 a, c. However, the average surface roughness values are much higher, with *S*_a_ between 0.2 µm and 0.4 µm across all experiments. One reason for this may be due to dry machining of Ti6Al4V material produces excessive friction and high cutting temperatures, leading to poor chip formation and ploughing mode of material removal, which is in line with the results presented on burr formation in the next section below. Overall, the results still indicate very little tool wear occurred for both diameter tools with relatively low surface roughness.

In contrast, very high overall surface roughness values were measured for 2-flute tools. This was especially true for CoCrMo workpiece material, where extremely high surface roughness values were measured for some experiments. The reason for this was that the 2-flute tools suffered major tool wear and complete tool failure during experiments on the CoCrMo workpiece, as can be seen in Figs. 14c, d. Excessive tool wear occurred during Exp 8 and progressed in Exp 9, i.e., Slots 8 and 9, for the 2-flute 800 µm tool experiments. This also occurred from Exp 6 through to Exp 9, for the 2-flute 600 µm tools. The excessive tool wear was also identified from the large cutting force for the same experiment numbers in Figs. 10 c, d. The overall surface roughness slightly increases with each experiment block, again indicating that significant tool wear is occurring and then progressing with each subsequent experiment after. Degradation of the cutting edge then leads to unstable machining conditions and poor surface quality. Surface roughness values are generally very high, with *S*_a_ > 0.2 µm in most experiments and up to *S*_a_ > 1 µm in some cases. Overall trends in this data are difficult to determine as a result of the excessive tool wear occurring inside each experiment block for both tool diameters. However, this was not the case during 2-flute experiments on the Ti6Al4V workpiece, as seen in Figs. 15c, d. Overall surface roughness generally increased with each experiment, indicating tool wear and higher feed rates within each block led to higher surface roughness values. This is similar to the surface roughness at the start of the slot increasing due to more unstable machining conditions at the start of the experiments. However, unlike the DSEE tools, the surface roughness at the end of the slots is significantly worse for 2-flute tools, which indicates that tool wear during machining is the most important factor that is affecting surface roughness in these experiments. This result is common across both 2-flute tool diameters and workpiece material.

#### Burr formation

**Fig. 16:**
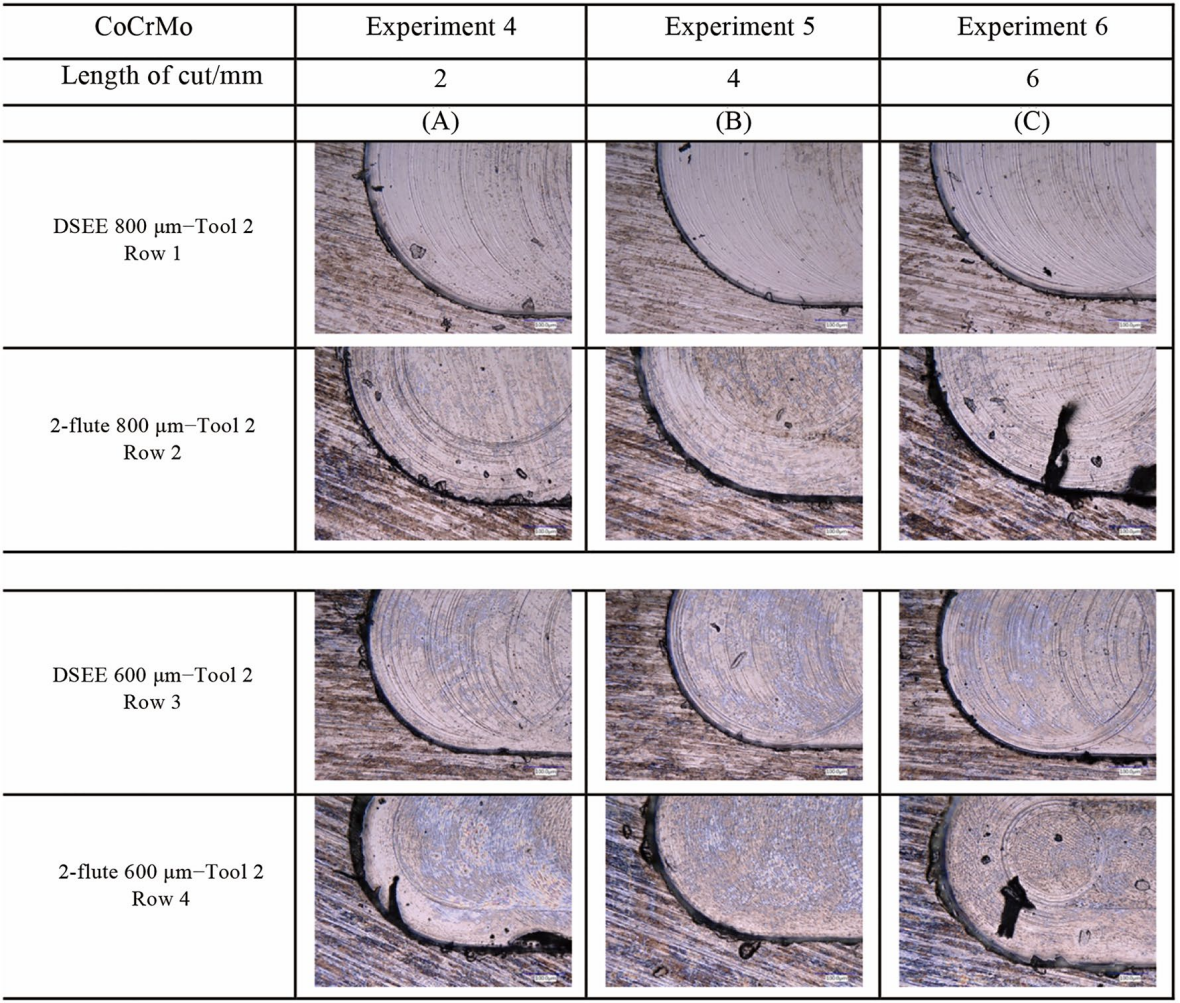
Burr formation at end of slot for both tool types and diameters for CoCrMo workpiece material

**Fig. 17:**
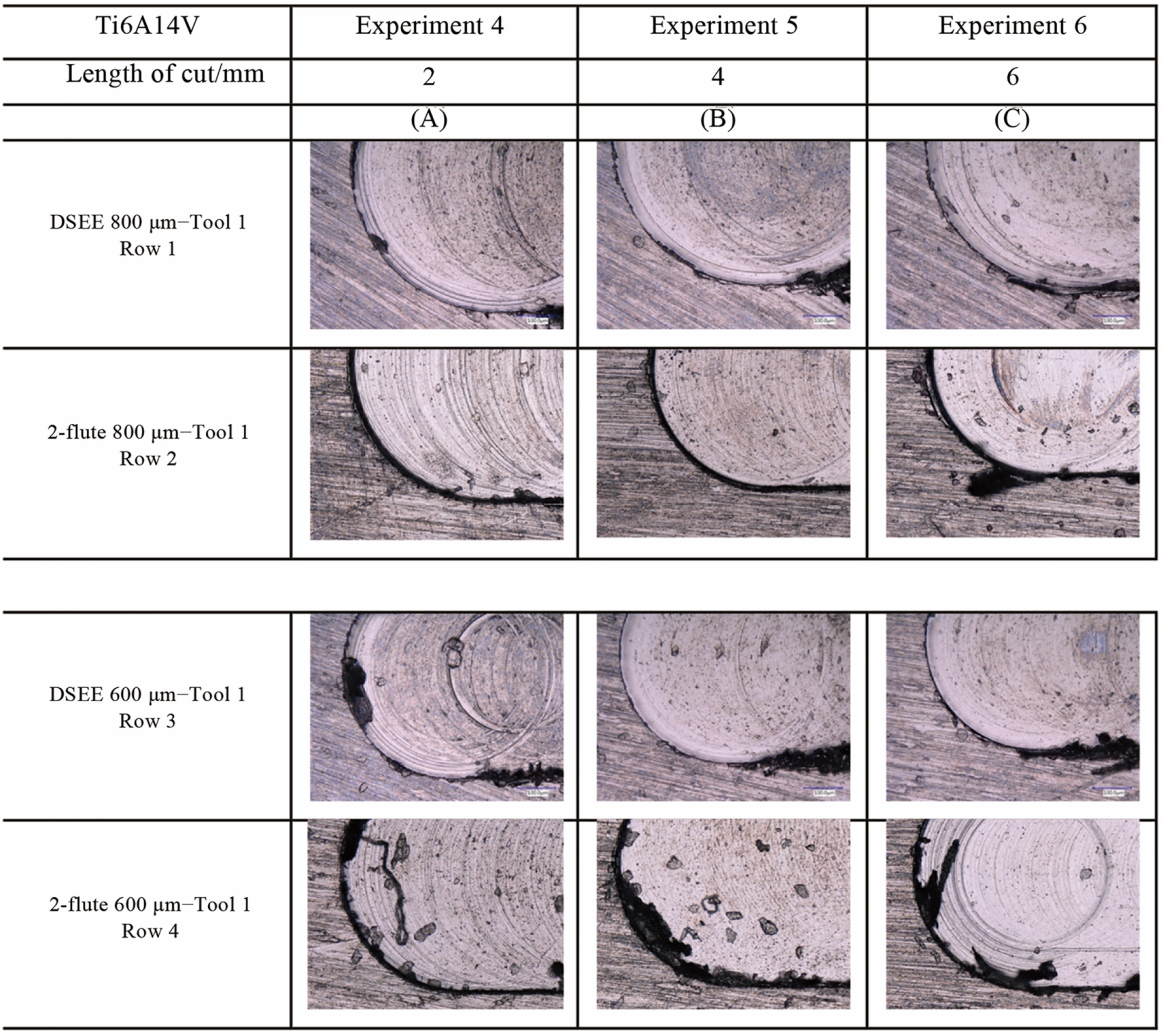
Burr formation at end of slot for both tool types and diameters for Ti6Al4V workpiece material

Overall, DSEE tools generally showed straight walls with little burr formation, while 2-flute tools generally showed rougher walls with large burr formation. Examples of the qualitative analysis on the machined slots are presented in Fig. 16 for CoCrMo and Fig. 17 for Ti6Al4V workpieces, respectively. Beginning with CoCrMo and Exp 4 for both tool diameters, sharp corners with minor burr formation are presented in Fig. 16a. Figure 16b provided the least amount of burr formation and sharpest edges, while Fig. 16c provided the most burrs and worst slot edges. As the tool is used for three experiments, therefore 6 mm cutting length total, tool wear has an effect on the quality of the side walls. However, it can be determined that feed rate has a more significant effect on machined slot edges, as only very low tool wear occurs for DSEE tools as has been previously shown. This is not the case for 2-flute tools which degrade quickly due to tool wear and therefore feed rate has a similar effect on tool wear burr formation. The overall trends are similar for 2-flute diameter tools, with very large burrs formed, damaged side walls and rounded corners for all three experiments. Figure 16c again provided the worst surface quality in this regard. Large, continuous chips can be seen still connected to the wall for both tool diameters for Fig. 16c. Lack of cutting fluid also had a major impact on burr formation due to high cutting temperatures caused by friction between the tool and workpiece.

The results of Ti6Al4V material are much poorer for both tool types and diameters. Larger and more regular burrs formed on the side wall in the same locations for each experiment, as can be seen in Fig. 17. In general, DSEE 800 µm tools resulted in larger burr formation on Ti6Al4V workpiece material than CoCrMo and more rounded wall edges. However, as the feed rate increased from Exps 4–6, the size and consistency of burrs formed were reduced, indicating higher feed rates were more optimal for this tool to increase slot quality. These results are consistent across both tool diameters. DSEE 600 µm tools provided relatively sharp slot corners, however produced more build-up of material and burrs formed at the end of the slot than the 800 µm tools. Both 2-flute diameter tools provided very poor slot quality across all experiments, resulting in large amounts of burr formation, uncut chips and continuous chip build-up on the side walls as well as burrs on the top surface. In the case for both 800 µm and 600 µm tools, feed rate had a negative impact on surface quality which indicated tool chipping, tool wear and tool breakage all occurred as the tool was too fragile for the higher feed rates necessary for machining this material. Similarly, as tool wear progressed and tool chipping worsened with higher feed rates, more ploughing and build-up of uncut chips occurred along the sidewall as tool cutting edge conditions worsened.

#### Chip size

**Fig. 18:**
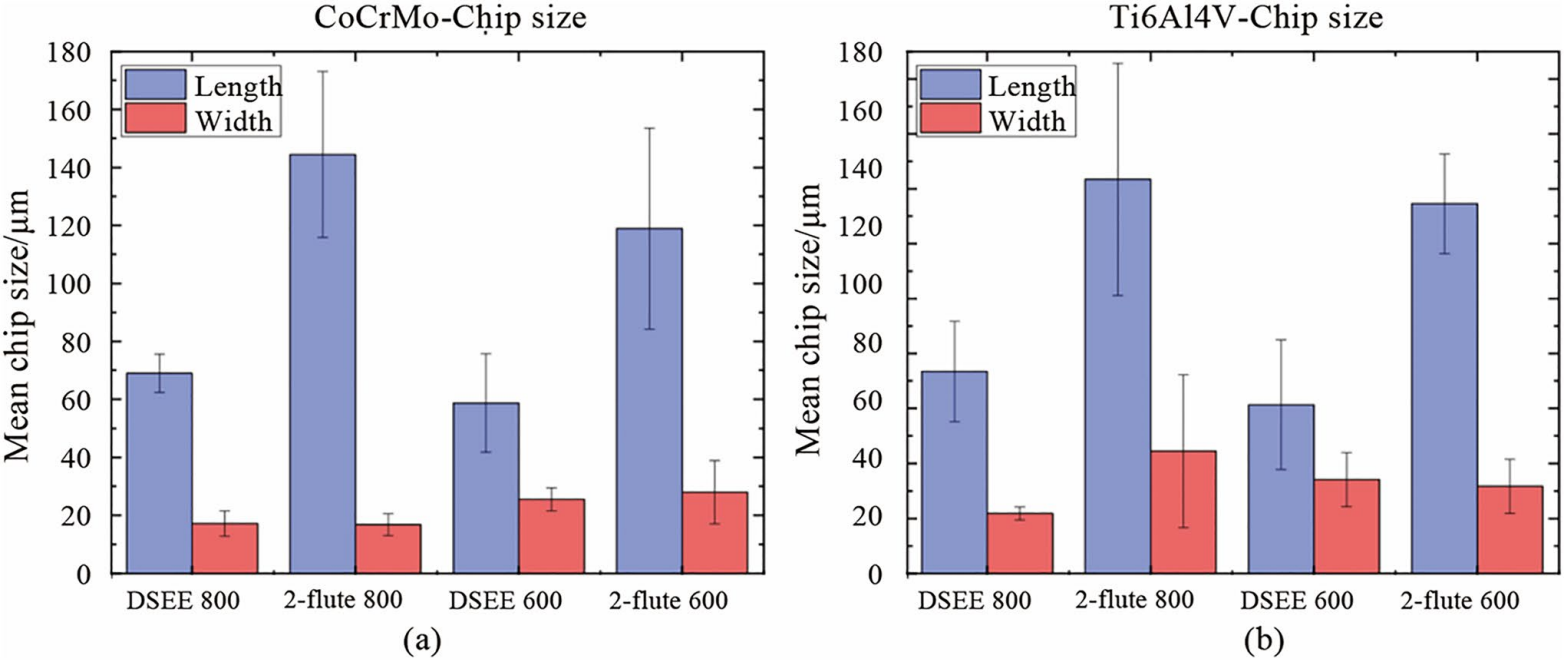
Chip length and width dimensions of chips formed with both tool types and diameters for **a** CoCrMo workpiece material and **b** Ti6Al4V

**Fig. 19:**
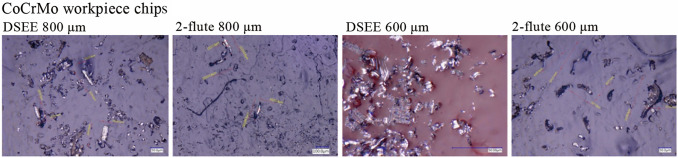
Chip size and shape measurements of CoCrMo for both tool types and diameter using ×2 000 magnification on a Keyence VHX-5000 digital microscope

**Fig. 20:**
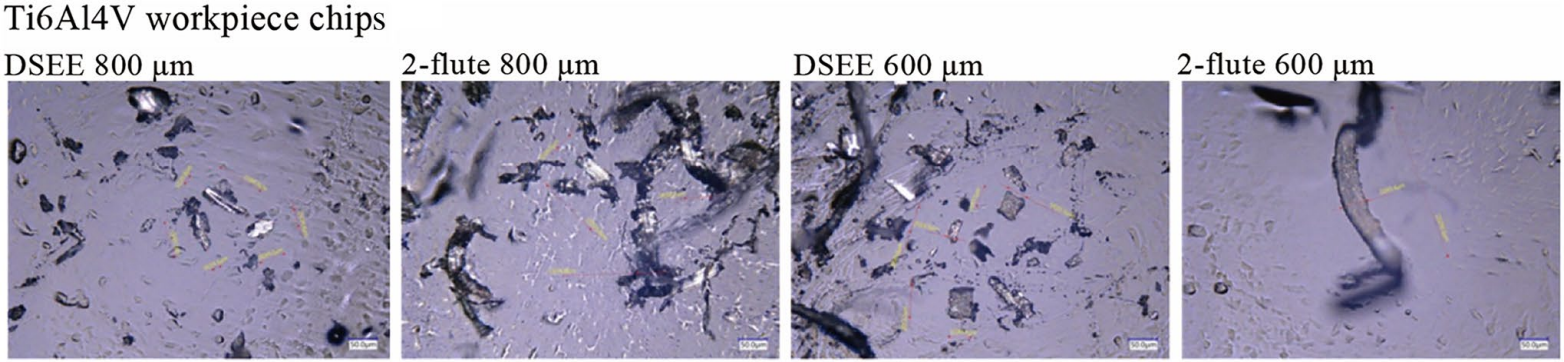
Chip size and shape measurements of Ti6Al4V for both tool types and diameter using × 2 000 magnification on a Keyence VHX-5000 digital microscope

The results of chip size and shape analysis are presented in Figs. 18−20, respectively. Figure 18 shows the mean chip size of both tool types and diameters in relation to length and width, for CoCrMo and Ti6Al4V. Beginning with chip dimensions of CoCrMo, the DSEE 800 µm produced short chip lengths and widths, with mean chip sizes of 69.0 µm and 17.18 µm, respectively. The DSEE 600 µm tool also produced small chips, with mean sizes of 58.68 µm and 25.53 µm. In contrast, both the 2-flute diameters produced far larger chips, with length and width of 144.43 µm and 16.78 µm for 800 µm tool, and 118.88 µm and 28.0 µm for 600 µm tool. The results of the Ti6Al4V workpiece material are again extremely similar. The DSEE 800 µm produced short chips, with mean chip length and width of 73.43 µm and 21.8 µm, respectively, and the DSEE 600 µm tool produced chips of sizes 58.68 µm and 25.53 µm. Both the 2-flute diameters again produced far larger chips, with length and width of 143.42 µm and 44.48 µm for 800 µm tool, and 134.57 µm and 31.68 µm for 600 µm tool. Overall, the chip size of the Ti6Al4V material was slightly larger than the CoCrMo material, notably in the width dimension, due to the difference in hardness between the two. Ti6Al4V alloy has a hardness of 36 HRC [30], while CoCrMo alloy has a hardness of about 45 HRC [31]. Therefore Ti6Al4V material is more ductile and will generally form slightly more continuous chips.

Overall, the results indicated that shorter, more discontinuous chips were formed during machining by the DSEE tools, leading to better tool performance and lower cutting forces during machining, as verified by the cutting force results in Sect. 4.3.1. Shorter and broader chips were formed for both workpiece materials, and could be seen in Fig. 19 for CoCrMo and Fig. 20 for Ti6Al4V. The reason for the short and discontinuous chip formation of DSEE tools is twofold, and both are as a result of the tool design. The first is in relation to the straight cutting edges, where chip formation is directed radially inward towards the centre of rotation of the tool, instead of up and away from the workpiece, as with fluted tool designs. This has the impact of creating shorter and broader chips as the chip curls in on itself and breaks away as new chip is further directed inwards. Whereas fluted designs push the chip up and away from the workpiece along the flute, creating longer but narrower chips. These results are verified by the cutting force results, where the DSEE tools produce high infeed cutting forces as the tool is pulled into the workpiece in the feed direction by a broad chip forming, but have low thrust force and therefore shorter chips. In comparison, the 2-flute tools have far higher thrust force as the forming chip pulls away from the workpiece up along the flute producing longer chips, while the tool is also forced downwards into it. The second reason for the short and discontinuous chip formation is as a result of the chip breaker features on both the rake and flank faces, which encourage the forming chip to curl and round on itself which then split and break as further material is directed inwards. Both design features are substantiated by both tool diameters and workpiece materials.

#### Tool wear

**Fig. 21:**
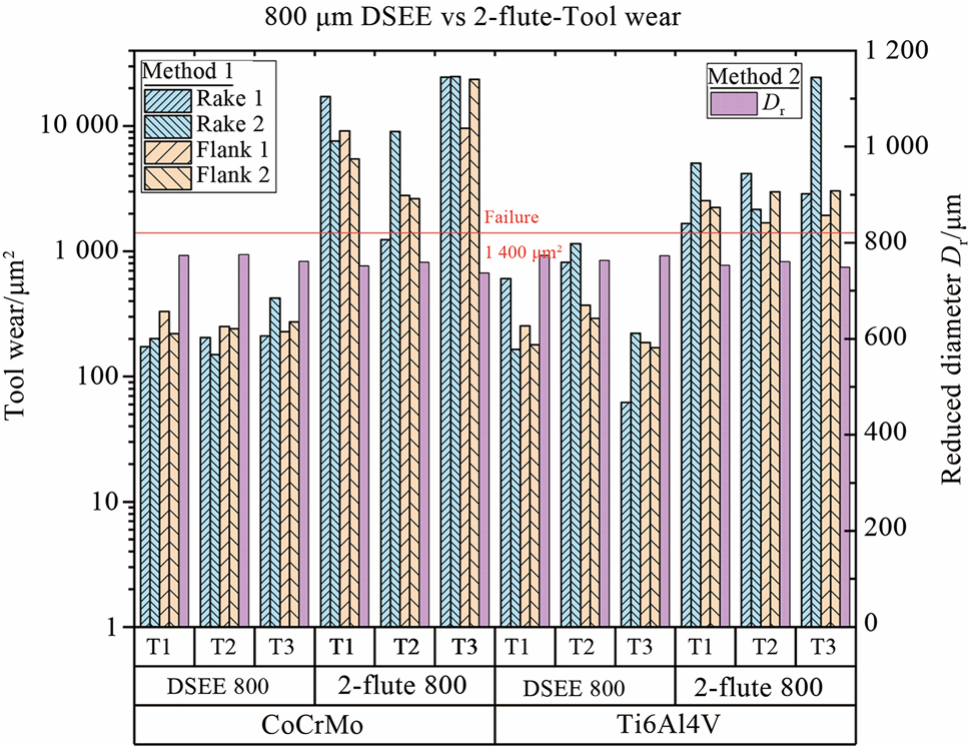
Measured tool wear for each tool (T(*x*)) for both tool types and workpiece materials for diameter 800 µm

**Fig. 22:**
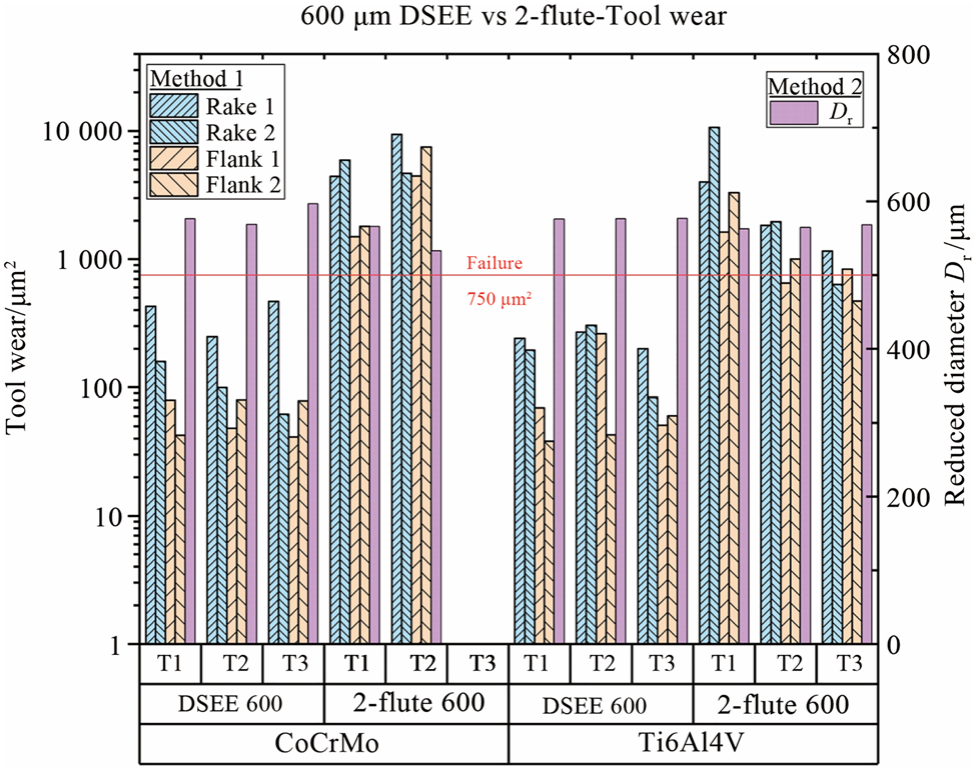
Measured tool wear for each tool (T(*x*) for both tool types and workpiece materials for diameter 600 µm

**Fig. 23:**
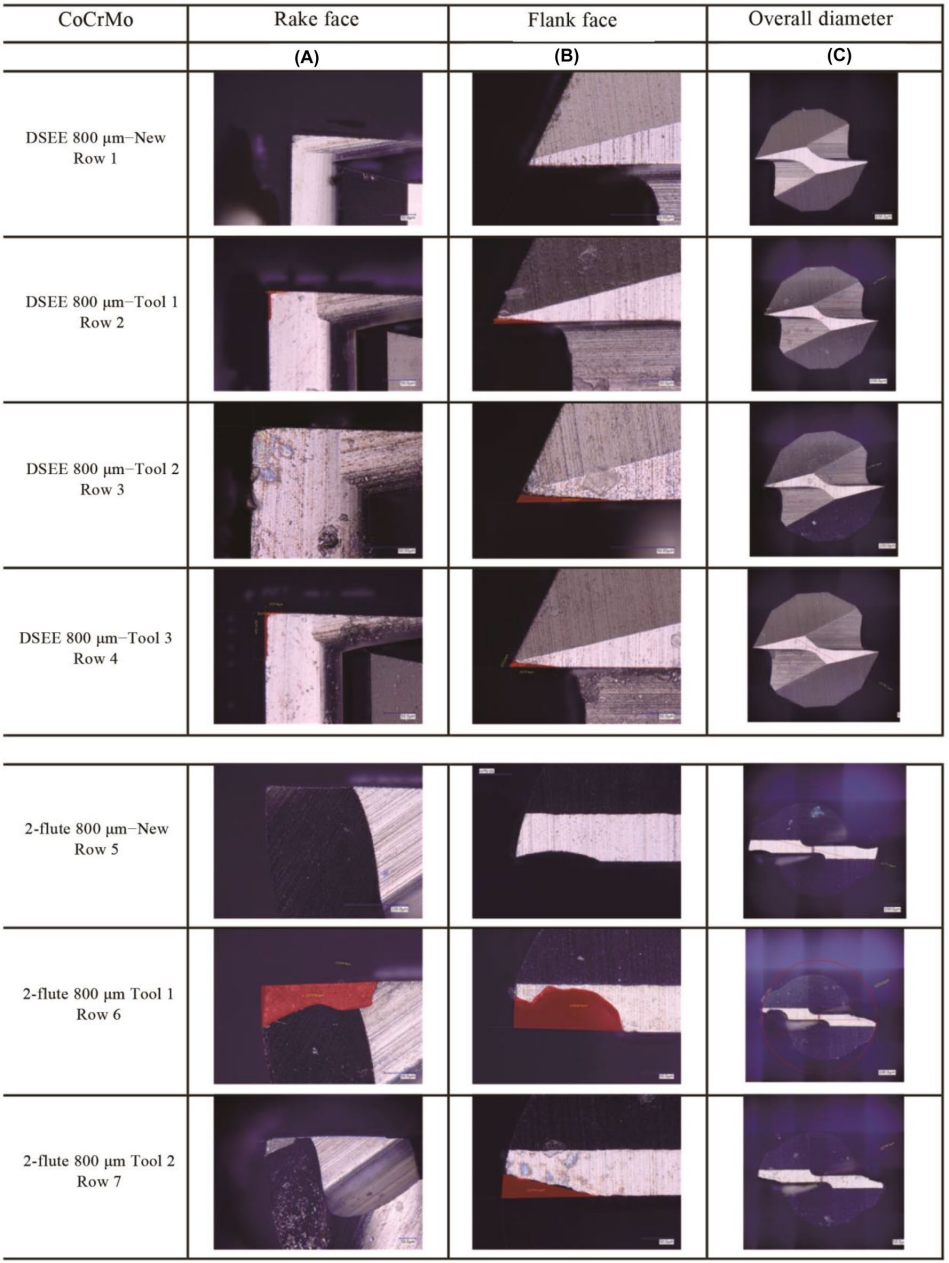
Determination of wear area on rake and flank faces, and reduced diameter of each 800 µm tool for CoCrMo workpiece material

The results of the tool wear analysis using the developed tool wear criterion are presented in Fig. 21 for both tool types and workpiece materials, representing 800 µm tools; and Fig. 22 represents 600 µm tools. Table 8 shows the typical results of both Method 1 and Method 2 analysis, with the tool wear area determined from Fig. 23. Only the 800 µm DSEE and 2-flute tools on CoCrMo workpiece are displayed in Tables 8, 9 and Fig. 23 for the sake of brevity. Beginning with CoCrMo workpiece and examining Fig. 21, all three DSEE 800 µm tools can still be considered “New” with little tool wear occurring in regards to both methods. Regarding Method 1, all three tools had relatively low rake and flank wear on both cutting edges, however Tool 3 had the highest rake face wear. This is in line with the infeed and crossfeed cutting forces and surface roughness data for this tool block, Exps 7–9, which are the highest for these experiments. Significant runout between rake faces is also evident in this tool, 211.4 µm^2^ and 422.5 µm^2^. This demonstrates that this set of experiments, Block 3, provided the worse process outputs and that the machining parameters were too high. Tool 2 had the lowest rake and flank face tool wear, with no significant runout or chipping occurring. Therefore, Block 2 of experiments provided the most optimal machining conditions. Tool 1 had overall low tool wear again, although chipping was identified on flank face 1. For Method 2, all three tools could be still be considered “New”, as no significant diameter reduction occurred and slot geometrical integrity was maintained, although Tool 3 was very close to be considered “Minor” wear. Conversely, the results for 2-flute 800 µm tool show “Complete Failure” occurred for each face of each flute in relation to Method 1 analysis. Tool 3 suffered the most tool wear for both flutes, and for both rake and flank faces, while Tool 2 offered the relatively lowest wear, agreeing with results that Block 2 had the most optimal machining parameters for CoCrMo. Analysis of Method 2 result shows only minor and major reduction of diameter occurred for Tools 1 and 2, respectively. Even though the rake and flank face cutting edges were completely destroyed, the outer diameter was still maintained. Therefore it is important to evaluate the tool wear in regards to both methods to better evaluate the overall condition of each tool.

**Table 8:** Method 1 and Method 2 characterisation of tool wear for 800 µm DSEE on CoCrMo workpiece (Values are determined from Fig. 23)

Tool No.	Rake 1 µm^2^	Rake 2 µm^2^	Average µm^2^	Method 1						Method 2	
				Condition	Flank 1 µm^2^	Flank 2 µm^2^	Average µm^2^	Condition	Other	*D*_r_ µm^2^	Condition
1	172.7	201.3	187.0	New	330.4	220.4	275.4	New	Chip	773.6	New
2	204.8	149.6	177.2	New	250.8	240.1	245.5	New	–	775.5	New
3	211.4	422.5	317.0	New	227.4	273.4	250.4	New	Runout	761.1	New

**Table 9:** Method 1 and Method 2 characterisation of tool wear for 2-flute tools on CoCrMo workpiece (Values are determined from Fig. 23)

Tool No.	Rake 1 µm^2^	Rake 2 µm^2^	Average µm^2^	Method 1						Method 2	
				Condition	Flank 1 µm^2^	Flank 2 µm^2^	Average	Condition	Other	*D*_r_ µm^2^	Condition
1	17 138	7 579.0	12 358.7	Complete Failure	8 760.9	5 075.4	6 918.2	CF	Broken	751.6	Minor/Major
2	1 238.2	9 023.0	5 130.6	Complete Failure	2 393.1	2 234.6	2 313.9	CF	Broken	759.3	Minor
3	24 528	24 937	24 732.7	Complete Failure	9 187.6	–	9 187.6	CF	Broken	–	CF

Comparing the results for both 600 µm tools for CoCrMo workpiece material in Fig. 21, more significant wear occurred for the DSEE 600 µm tool than for DSEE 800 µm tool. “Minor” rake face wear occurred on all three DSEE 600 µm tools while runout was evident when comparing both rake face cutting edges in Method 1. Very little flank face wear occurred over each tool. Again, Tool 2 provided the lowest tool wear while Tools 1 and 3 were comparable. Tool condition was overall poorer when analysing Method 2 results, which indicated minor wear occurred over Tools 2 and 3 but not for Tool 1.In comparison, Method 1 results of 2-flute 600 µm tool show “Complete Failure” occurred for Tools 1 and 2, while no data could be collected for Tool 3 as it snapped and was destroyed, indicating the machining parameters in this block were too high for this tool. Method 2 results indicate only “Minor” and “Major” wear occurred for Tools 1 and 2, respectively. However in this case, Method 1 results present a more realistic case for tool condition.

Figure 21 also presents the results for DSEE and 2-flute 800 µm tools for workpiece material Ti6Al4V. Slightly more wear occurred for this material than for CoCrMO. Considering DSEE 800 µm results from Method 1, Tool 3 had the lowest rake and flank face wear and the tool can still be considered “New” after Exps 7–9, with minor chipping occurring. This indicates that higher feed rates and spindle speeds provide better machining characteristics for this material, reinforced by lower thrust and crossfeed cutting forces as well as comparable surface roughness results with the least amount of tool wear. Tool 2 provided the highest tool wear with “Major” rake face wear occurring. Method 2 agreed with these results and described Tool 3 condition as “New” and Tool 2 condition as “Minor”. Again, Method 1 determined that all 2-flute 800 µm tools were considered “Complete Failure” with no other information to offer due to destroyed cutting edges. In this case, Tool 3 had the worst tool condition which indicated that these tools could not achieve the high feed per tooth necessary for high material removal rates. Method 2 again gave poor information when rake and flank faces were completely destroyed.

Figure 22 displays the results for DSEE and 2-flute 600 µm tools for Ti6Al4V workpiece material. Considering the DSEE 600 µm tools, very little tool wear occurred. Again, Tool 3 provided the lowest tool wear conditions for both rake and flank faces while Tool 2 was considered “Minor” tool wear for both rake and flank faces, with slight runout identified. Therefore Block 3 provided the best machining parameters for this workpiece material. Method 2 analysis considers all three tools to be “New”, although they are all borderline “Minor” wear, which gives good information on the overall cutting diameter and they should be monitored before “Major” wear occurs, to prevent geometrical errors of the machined feature. The results of Method 1 analysis for the 2-flute 600 µm tools show “Complete Failure” of Tools 1 and 2 occurred, while Tool 3 suffered “Major” and “Minor” wear on rake and flank faces, respectively. This indicates that Block 3 machining parameters provide better machining characteristics again, in line with results from DSEE 800 µm and 600 µm results. This also aligns with the results of both cutting force and surface roughness results, above, for Exps 7–9. Finally, Method 2 analysis results indicate that only “Minor” wear occurred to all three tools, which does not present an accurate reflection on the state of tool wear that occurred.

## Conclusions

A micro-milling DSEE tool design was proposed and achieved for machining of very hard and wear resistant materials with the purpose of significantly reducing tool wear and ensuring high quality machined slots. To fulfil such stringent requirements, the tool has met all the criteria proposed in the design specifications, namely, high stiffness, high strength, high durability, simplified and radially symmetric geometry and efficient chip evacuation properties.The main conclusions from this study can be drawn as follows.

(i) The DSEE tool shows overall much lower cutting forces than the 2-flute tools for the two types of workpiece materials. Crossfeed cutting force is slightly higher for DSEE tools than for 2-flute tools while thrust force is far lower, providing more stable machining conditions and better surface quality. The cutting force also increases with feed rate, per block of spindle speeds, and that higher spindle speed blocks with higher feed rates also result in higher cutting force.
(ii) Experiments with the DSEE tools produced much lower surface roughness values than the 2-flute design over both workpiece materials. Feed rate had only minor effect on the surface roughness, with the lowest *S*_a_ values occurring for feed per tooth of 0.4 µm for both tool diameters. Slightly lower surface roughness at the end of the slots show that very minor tool wear reduced the tool edge radius and removed sharp edges of the DSEE tool, thereby improving surface roughness values at the end of the slot. However, major tool chipping and tool breakage throughout 2-flute experiments resulted in very high surface roughness values. Little variation in surface roughness over the entire slots machined by DSEE tools indicate that the tool remained in good condition without edge chipping throughout each experiment block.
(iii) Burr formation was far lower for DSEE tools, while sharp slot edges were formed on CoCrMo workpiece material. 2-flute tools generally showed rougher and damaged side walls with large burr formation. Exp 5 again provided the most optimal machining conditions for this material. Burr formation was more significant on Ti6Al4V material for both tool types, however the DSEE tool showed far better results as feed per tooth increased, which indicated higher feed rates and spindle speeds were necessary for micro-milling with this material.
(iv) Shorter and more discontinuous chips were formed during machining by the DSEE tools, while the 2-flute tools produced longer and more continuous chips. DSEE tools reduced the length of the chip by almost half in comparison to 2-flute tools, while only slightly increasing chip width.
(v) All DSEE tools sustained very little tool wear throughout the experiments, with most tools still being considered “New”, according to both analysis methods of the developed tool wear criterion. Based on the experimental results, the conventional 2-flute tool design is not capable of micro-milling very hard materials and is prone to the major issues of massive tool wear, tool breakage and edge chipping when machining.
